# STAT5 is a key transcription factor for IL-3-mediated inhibition of RANKL-induced osteoclastogenesis

**DOI:** 10.1038/srep30977

**Published:** 2016-08-03

**Authors:** Jongwon Lee, Semun Seong, Jung Ha Kim, Kabsun Kim, Inyoung Kim, Byung-chul Jeong, Kwang-Il Nam, Kyung Keun Kim, Lothar Hennighausen, Nacksung Kim

**Affiliations:** 1 Department of Pharmacology, Chonnam National University Medical School, Gwangju 61469, Republic of Korea; 2 Department of Biomedical Sciences, Chonnam National University Medical School, Gwangju 61469, Republic of Korea; 3 Department of Anatomy, Chonnam National University Medical School, Gwangju 61469, Republic of Korea; 4 Laboratory of Genetics and Physiology, National Institute of Diabetes and Digestive and Kidney Diseases, National Institutes of Health, Bethesda, MD 20814, USA.

## Abstract

Among the diverse cytokines involved in osteoclast differentiation, interleukin (IL)-3 inhibits RANKL-induced osteoclastogenesis. However, the mechanism underlying IL-3-mediated inhibition of osteoclast differentiation is not fully understood. Here we demonstrate that the activation of signal transducers and activators of transcription 5 (STAT5) by IL-3 inhibits RANKL-induced osteoclastogenesis through the induction of the expression of *Id* genes. We found that STAT5 overexpression inhibited RANKL-induced osteoclastogenesis. However, RANKL did not regulate the expression or activation of STAT5 during osteoclast differentiation. STAT5 deficiency prevented IL-3-mediated inhibition of osteoclastogenesis, suggesting a key role of STAT5 in IL-3-mediated inhibition of osteoclast differentiation. In addition, IL-3-induced STAT5 activation upregulated the expression of *Id1* and *Id2*, which are negative regulators of osteoclastogenesis. Overexpression of ID1 or ID2 in STAT5-deficient cells reversed osteoclast development recovered from IL-3-mediated inhibition. Importantly, microcomputed tomography and histomorphometric analysis revealed that STAT5 conditional knockout mice showed reduced bone mass, with an increased number of osteoclasts. Furthermore, IL-3 inhibited RANKL-induced osteoclast differentiation less effectively in the STAT5 conditional knockout mice than in the wild-type mice after RANKL injection. Taken together, our findings indicate that STAT5 contributes to the remarkable IL-3-mediated inhibition of RANKL-induced osteoclastogenesis by activating *Id* genes and their associated pathways.

Proper development and activity of osteoblasts and osteoclasts, which is responsible for bone formation and resorption, respectively, is crucial for healthy bone homeostasis. An imbalance between the numbers of the two cell types causes pathological problems such as osteopetrosis or osteoporosis, and many studies have aimed to understand the molecular mechanisms underlying osteoblast/osteoclast development and function.

Osteoblasts originate from mesenchymal stem cells, whereas osteoclasts differentiate from monocyte/macrophage lineage cells originating from hematopoietic stem cells. In *in vitro* culture systems, macrophage-colony stimulating factor (M-CSF) and receptor-activated nuclear factor kappa-B ligand (RANKL) can sufficiently aid in the development of osteoclasts^1^. *In vivo* studies revealed that RANKL overexpression causes osteoporosis. Conversely, RANKL knockout mice showed a striking osteopetrotic phenotype *in vivo*. Upon RANKL stimulation, RANK (RANKL receptor) recruits the adaptor protein TRAF6 and signal cascades are activated via the activation of signaling molecules such as ERK, JNK, P38 MAPK, and NF-κB^2^,^3^. Furthermore, RANKL increases the expression of the early osteoclastic gene, *c-Fos*, which in turn upregulates a master regulator of osteoclastogenesis, NFATc1^4^. NFATc1 can cooperate with other transcription factors such as PU.1 and Mitf, resulting in the induction of the expression of osteoclastic genes including *Oscar*, *Acp5*, *Ctsk*, and *Atp6v0d2*^5^,^6^,^7^.

Osteoclasts have been reported to be closely related to the immune system^7^. Osteoclast differentiation and function can be affected not only directly by immune cells but also indirectly via many cytokines produced by immune cells or other cell types. Pro-resorptive cytokines such as interleukin-1 (IL-1), TNF-α, and IL-17 strongly induce osteoclast formation and function. T cells produce various cytokines including interferon (IFN)-γ, IL-3, IL-4, and IL-10, which exert potent inhibitory effects on osteoclast differentiation^8^. IL-3, a cytokine secreted predominantly by activated T lymphocytes, serves as a link between the immune system and the hematopoietic system. Although studies using either organ culture or whole bone marrow cultures have revealed IL-3 to be a stimulator of osteoclastogenesis *in vitro*^9^,^10^, recent studies have shown that IL-3 irreversibly inhibits RANKL-induced osteoclast differentiation by downregulation of c-Fos expression, prevention of NF-κB signaling, and inhibition of RANK expression^11^,^12^. In addition, inhibitors of differentiation and DNA binding (Ids) are involved in the inhibition of RANKL-induced osteoclast differentiation and are known to be induced by IL-3. Thus, IL-3 negatively regulates osteoclast differentiation by regulating Ids and c-Fos expression^13^,^14^.

Signal transducers and activators of transcription (STATs) are a family of latent cytoplasmic proteins that are activated to participate in gene control when cells encounter various cytokines^15^. Seven known mammalian STAT proteins such as STAT1, 2, 3, 4, 5A, 5B, and 6 exist. STATs are activated by tyrosine phosphorylation following the binding of specific ligands to cognate receptors, leading to dimerization and subsequent translocation of the STATs to the nucleus^15^. Activated STATs bind to specific DNA motifs in regulatory regions and thereby control the transcription of genes that regulate cell proliferation, differentiation, apoptosis, and immune responses.

Two members of the STAT family, STAT5A and STAT5B (collectively called STAT5), have gained prominence owing to the fact that they are activated by a wide variety of cytokines such as IL-3 and granulocyte-macrophage colony-stimulating factor (GM-CSF), which are known to play important roles in the growth and differentiation of hematopoietic precursors^16^,^17^,^18^. Emerging evidence suggests that the STAT signaling pathway plays an important role in bone development and metabolism^19^. Recently, it was demonstrated that STAT5 negatively regulates bone resorption of osteoclasts by inducing Dusp gene expression^20^. However, although STAT5 has been shown to play a role in bone metabolism, the contribution of STAT5 to osteoclast differentiation has not yet been revealed.

In this study, the role of STAT5 in osteoclast differentiation and the underlying mechanism was investigated. The effect of IL-3 on osteoclast development were investigated in Mx1-Cre transgenic mice in which floxed Stat5 was specifically deleted. We discovered that the inhibitory effect of IL-3 on RANKL-induced osteoclastogenesis was dependent on STAT5 activation. In addition, STAT5 activation induced *Id* gene expression, resulting in the inhibition of osteoclast differentiation. Thus, we concluded that STAT5 inhibits osteoclast differentiation by controlling negative regulators upon IL-3 stimulation.

## Results

### STAT5 activation attenuates RANKL-induced osteoclast differentiation

**Figure 1:**
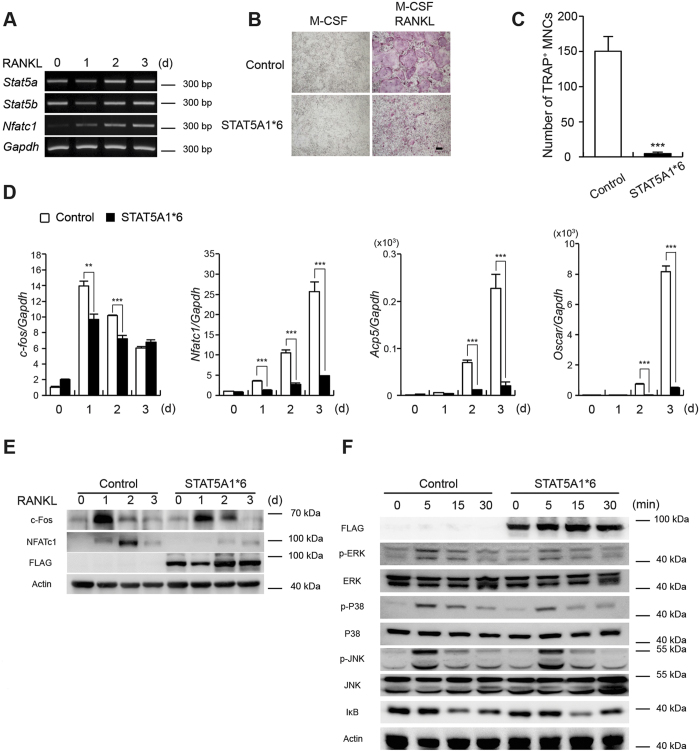
STAT5A activation reduces RANKL-mediated osteoclastogenesis. (**A**) BMMs were cultured with M-CSF and RANKL for the indicated times. Total RNA was collected at each time point. RT-PCR was performed to detect expression of the indicated genes. All gels run under the same experimental conditions and the representative images were cropped and shown. (**B**,**C**) BMMs were transduced with pMX-IRES-EGFP (control) or constitutively active STAT5A (STAT5A1*6) retrovirus and cultured with M-CSF in the presence or absence of RANKL for three days. (**B**) Cultured cells were stained for TRAP. (**C**) The number of TRAP-positive MNCs per well was counted. Data are represented as the mean ± SD. ***P < 0.001 vs. control; n = 4. (**D–F**) BMMs were transduced with pMX-IRES-EGFP (control) or STAT5A1*6 retrovirus and cultured with M-CSF and RANKL for the indicated times. (**D**) mRNA levels of *c-fos, Nfatc1*, *Acp5* and *Oscar* were assessed by quantitative real-time PCR. Data represent mean ± SD of triplicate samples. **P < 0.01; ***P < 0.001 vs. control. (**E**,**F**) Whole cell lysates were harvested from cultured cells and were immunoblotted with the indicated antibodies. All gels run under the same experimental conditions and the representative images were cropped and shown. Bar: 100 μm. All results are representative of at least three independent experiments. Statistical analyses were implemented in TTEST.

To understand the role of STAT5 in osteoclast differentiation, we initially examined *Stat5* levels during RANKL-induced osteoclastogenesis using RT-PCR. When bone marrow-derived macrophage-like cells (BMMs) were cultured with M-CSF and RANKL, the expression of NFATc1, a master transcription factor for osteoclast differentiation, strongly increased. The mRNA expression of *Stat5a* and *Stat5b* was observed throughout osteoclastogenesis (Fig. 1A). Next, the effect of STAT5 on osteoclast differentiation was examined by overexpressing a constitutively active form of STAT5A (STAT5A1*6) in osteoclast precursors. STAT5A1*6 overexpression strongly attenuated RANKL-induced osteoclastogenesis (Fig. 1B) and resulted in a significant reduction in the number of TRAP-positive multinucleated cells (Fig. 1C). Consistently, the expression of RANKL-inducible osteoclastic genes including *c-fos*, *Nfatc1*, *Acp5*, and *Oscar* was significantly reduced by ectopic STAT5A1*6 expression (Fig. 1D). Correspondingly, c-Fos and NFATc1 protein levels were also decreased (Fig. 1E). Moreover, activated STAT5 attenuated pERK and p38 levels in the early stages of RANKL-induced osteoclast differentiation (Fig. 1F). These results suggest that active STAT5A inhibits osteoclastogenesis, which is accompanied by the reduced expression of genes associated with osteoclast differentiation.

### RANKL-induced osteoclast differentiation is not affected by STAT5 deficiency

**Figure 2:**
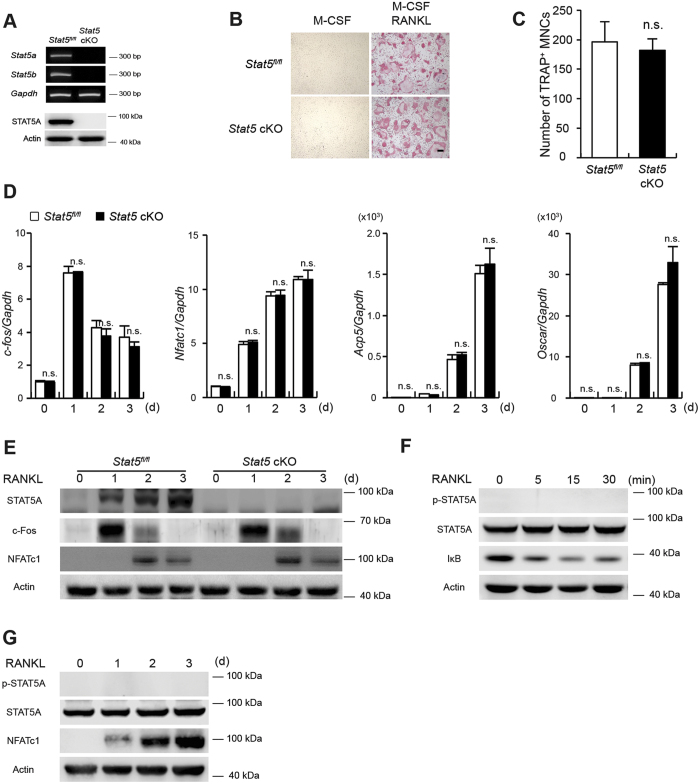
STAT5 deficiency does not regulate osteoclastogenesis. (**A**–**E**) Bone marrow cells were harvested from long bones of STAT5 conditional knockout (*Stat5* cKO) mice or *Stat5*^*fl/fl*^ littermates. (**A**) Total RNA was isolated from BMMs and RT-PCR was performed to detect expression of the indicated genes (upper panel). Whole cell lysates were harvested from BMMs and were immunoblotted with antibodies against STAT5A or actin (lower panel). All gels run under the same experimental conditions and the representative images were cropped and shown. (**B**,**C**) BMMs were cultured with M-CSF in the presence or absence of RANKL for three days. (**B**) Cultured cells were stained for TRAP. (**C**) The number of TRAP-positive MNCs per well was counted. Data are represented as the mean ± SD. n.s., not significant; n = 4. (**D**,**E**) BMMs were cultured with M-CSF and RANKL for the indicated times. (**D**) mRNA levels of *c-fos, Nfatc1*, *Acp5* and *Oscar* were assessed by quantitative real-time PCR. Data represent mean ± SD of triplicate samples. n.s., not significant. (**F**) BMMs were stimulated with RANKL for the indicated times. (**G**) BMMs were cultured with M-CSF and RANKL for the indicated times. (**E**–**G**) Whole cell lysates were harvested from cultured cells and were immunoblotted with the indicated antibodies. All gels run under the same experimental conditions and the representative images were cropped and shown. Bar: 100 μm. All results are representative of at least three independent experiments. Statistical analyses were implemented in TTEST.

Osteoclast differentiation in the absence of STAT5 was examined in cells obtained from MX1-Cre recombinase-mediated *Stat5* conditional knockout (cKO) mice, targeting both STAT5 isoforms in osteoclasts. We first confirmed the almost complete absence of *Stat5a* and *Stat5b* mRNA and STAT5 protein in the BMMs (Fig. 2A). Unexpectedly, upon differentiation from BMMs lacking STAT5, osteoclast formation was unimpaired and comparable with that observed with *Stat5*^*fl/fl*^ cells (Fig. 2B,C). Furthermore, the mRNA levels of *c-fos*, *Nfatc1*, *Acp5*, and *Oscar* were not altered by the absence of STAT5 (Fig. 2D). In addition, c-Fos and NFATc1 protein levels were comparable between the *Stat5*^*fl/fl*^ and *Stat5* cKO samples (Fig. 2E). These data suggested the possibility that RANKL signaling does not regulate STAT5 expression. We therefore examined STAT5 phosphorylation upon RANKL stimulation. Upon the stimulation of BMMs with RANKL for a short period of time following starvation, no STAT5 phosphorylation was observed at any time over the duration of RANKL stimulation, whereas IκB degradation was clearly evident (Fig. 2F), indicating that BMMs had been sufficiently stimulated by RANKL. Furthermore, STAT5 phosphorylation was never observed during RANKL-mediated osteoclastogenesis, whereas NFATc1 expression was strongly induced during osteoclast differentiation (Fig. 2G). In summary, these data show that STAT5 deficiency did not alter RANKL-induced osteoclast differentiation, suggesting that STAT5 activation does not occur via the RANKL signaling pathway.

### IL-3 attenuates RANKL-induced osteoclast differentiation

Although STAT5 activation is not regulated by RANKL, it did result in significant suppression of osteoclast differentiation. To address this conundrum, we searched for other cytokines capable of activating STAT5 and thereby inhibiting RANKL-induced osteoclast differentiation. IL-3 has been shown to activate STAT5 in a variety of cells^21^, including by BMMs, as shown in this study (Supplementary Fig. 1A). We observed osteoclast differentiation in the presence of increasing concentrations of IL-3 from 10 pg/mL to 1 ng/mL; IL-3 also suppressed RANKL-induced osteoclast differentiation in a dose-dependent manner (Supplementary Fig. 1B,C). Thus, a negative effect of IL-3 on osteoclast development would be consistent with reports of STAT5-mediated inhibition of osteoclastogenesis.

### STAT5 deficiency prevents IL-3-mediated inhibition of osteoclastogenesis

**Figure 3:**
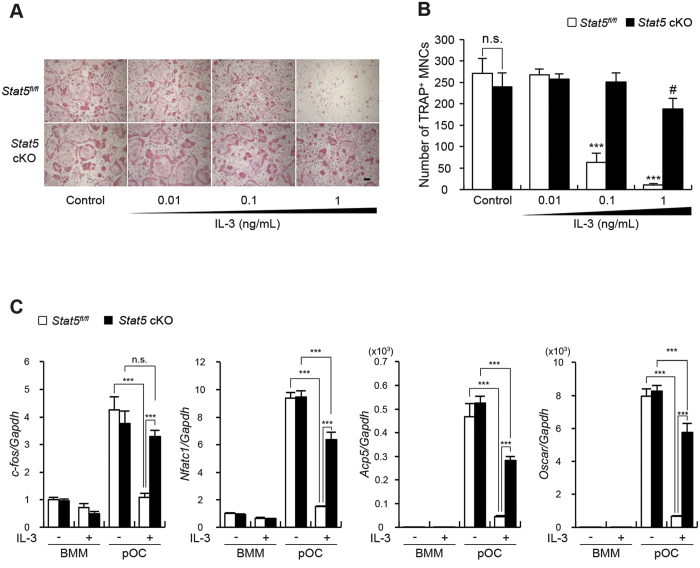
IL-3 inhibits RANKL-mediated osteoclastogenesis in a STAT5-dependent manner. Bone marrow cells were harvested from long bones of *Stat5* cKO mice or *Stat5*^*fl/fl*^ littermates. (**A**,**B**) BMMs were cultured with M-CSF and RANKL for three days in various concentrations of IL-3. (**A**) Cultured cells were stained for TRAP. (**B**) The number of TRAP-positive MNCs per well was counted. Data are represented as the mean ± SD. ***P < 0.001 vs. *Stat5*^*fl/fl*^ control; ^#^P < 0.05 vs. *Stat5* cKO control; n.s., not significant; n = 4. (**C**) BMMs were treated with IL-3 (1 ng/mL). Cells were further cultured in the presence (pOC) or absence (BMM) of RANKL for two days, and subjected to semi-quantitative real-time PCR for the indicated genes. Data represent mean ± SD of triplicate samples. ***P < 0.001; n.s., not significant. Bar: 100 μm. All results are representative of at least three independent experiments. All data were analyzed using ANOVA.

Next, we determined whether the inhibitory effect of IL-3 on osteoclast differentiation is attributable to STAT5 activation. To this end, we tested whether the inhibitory effect of IL-3 on osteoclast differentiation was abrogated by STAT5 deficiency. When osteoclast differentiation from *Stat5*^*fl/fl*^ cells and *Stat5* cKO cells in the presence of IL-3 was compared, it was found that STAT5 deficiency partially restored osteoclast formation that was inhibited by IL-3 in the *Stat5*^*fl/fl*^ cells (Fig. 3A,B). This was confirmed by mRNA expression of the osteoclastic genes *c-fos*, *Nfatc1*, *Acp5*, and *Oscar*. In the *Stat5*^*fl/fl*^ cells, the expression of RANKL-inducible *c-fos*, *Nfatc1*, *Acp5*, and *Oscar* was strongly suppressed by IL-3 in pre-osteoclasts (pOC). In contrast, in *Stat*5 cKO cells, the expression of *c-fos* was recovered to almost the level observed in the absence of IL-3, while the expression of *Nfatc1*, *Acp5*, and *Oscar* was lower than that in the absence of IL-3. However, the expression was still higher than that in the IL-3–treated pOC from the *Stat5*^*fl/fl*^ cells (Fig. 3C). Taken together, these results strongly suggest that STAT5 is a key modulator of the IL-3-mediated inhibition of osteoclast development.

### Activation of STAT5A or STAT5B alone is sufficient to inhibit osteoclast differentiation

Since redundancy between STAT5A and STAT5B has been proposed in other cell types, we tested this possibility in osteoclastogenesis. Overexpression of either wild-type STAT5A or STAT5B caused a similar reduction in osteoclastogenesis, although the extent of inhibition was weaker than that observed upon overexpression of the constitutive active STAT5A1*6 (Supplementary Fig. 2A,B). In addition, downregulation of RANKL-mediated NFATc1 induction in osteoclastogenesis was also comparable when STAT5A and STAT5B were ectopically expressed, but this was significantly less than that observed in the control (Supplementary Fig. 2C). Furthermore, IL-3 inhibited osteoclast differentiation by approximately 23% and 30% following overexpression of STAT5A and STAT5B, respectively (Supplementary Fig. 2D,E), indicating overlapping functions of STAT5A and STAT5B in the inhibition of osteoclast differentiation. Since STAT5A and STAT5B exhibited an overlapping inhibitory effect on osteoclast differentiation with similar potentials, we determined whether the absence of either isoform would still inhibit RANKL-induced osteoclast differentiation. To address this, a constitutively active form of STAT5A (STAT5A1*6) was overexpressed in the BMMs obtained from *Stat5*^*fl/fl*^ and *Stat5* cKO mice. Expression of the activated STAT5A in *Stat5* cKO BMMs led to the attenuation of TRAP positive multi-nucleated cells to a degree comparable to that observed in the *Stat5*^*fl/fl*^ samples (Supplementary Fig. 3A,B). This provides compelling evidence indicating that activation of either isoform alone is sufficient to inhibit osteoclast differentiation.

### ID1 and ID2 are responsible for the STAT5-mediated inhibition of osteoclast differentiation

**Figure 4:**
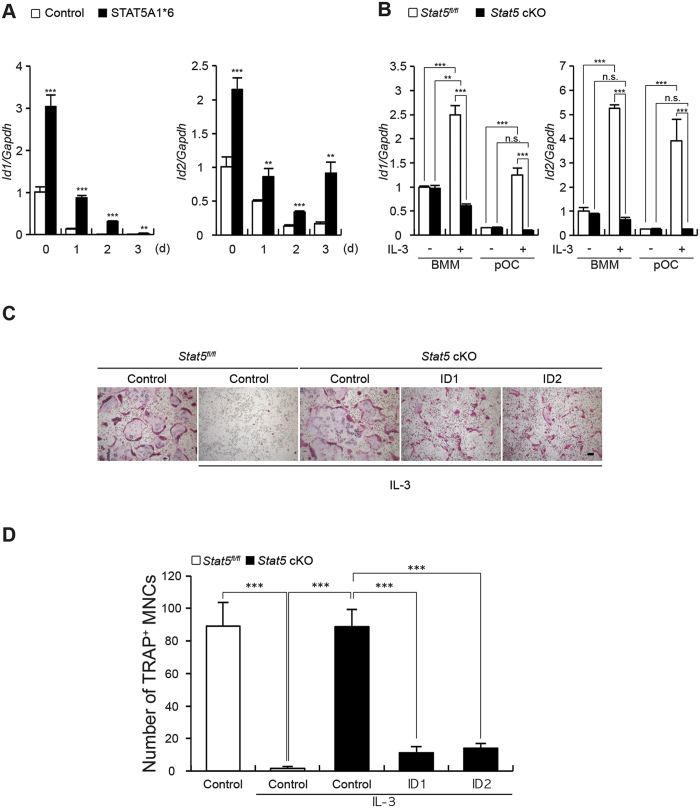
Id1 and Id2 are responsible for STAT5-mediated inhibition f osteoclastogenesis. (**A**) BMMs were transduced with pMX-IRES-EGFP (control) or STAT5A1*6 retrovirus and cultured with M-CSF and RANKL for the indicated times. Levels of *Id1* and *Id2* mRNA were assessed by quantitative real-time PCR. Data represent mean ± SD of triplicate samples. **P < 0.01; ***P < 0.001 vs. control. (**B**–**D**) Bone marrow cells were harvested from long bones of *Stat5* cKO mice or *Stat5*^*fl/fl*^ littermates. (**B**) BMMs were treated with IL-3 (1 ng/mL) and cells were further cultured in the presence (pOC) or absence (BMM) of RANKL for two days. Levels of *Id1* and *Id2* mRNA were assessed by quantitative real-time PCR. Data represent mean ± SD of triplicate samples. **P < 0.01; ***P < 0.001 vs. control; n.s., not significant. (**C**,**D**) Bone marrow cells were harvested from long bones of *Stat5* cKO mice or *Stat5*^*fl/fl*^ littermates. BMMs were transduced with pMX-IRES-EGFP (control), Id1 or Id2 retrovirus and cultured with M-CSF and RANKL for three days in the presence or absence of IL-3, as indicated. (**C**) Cultured cells were stained for TRAP. (**D**) The number of TRAP-positive MNCs per well was counted. Data are represented as the mean ± SD. ***P < 0.001 vs. control; n = 4. Bar: 100 μm. All results are representative of at least three independent experiments. Data were analyzed using TTEST (**A**) or ANOVA (**B**,**C**).

To understand the mechanism underlying the inhibition of osteoclast differentiation by STAT5, we searched for STAT5 target genes that were regulated during both RANKL and IL-3 stimulation. RNA sequencing was performed on the BMMs obtained from the *Stat5*^*fl/fl*^ and *Stat5* cKO mice stimulated with M-CSF and RANKL, in the presence or absence of IL-3 (GSE76988). The RNA sequencing data indicated that the mRNA expression of *Id1* and *Id2* was attenuated upon RANKL stimulation and that the levels were recovered by IL-3 stimulation, but not in the absence of STAT5 (Supplementary Table 1). Therefore, we proposed that *Id1* and *Id2* are the target genes of STAT5. It has also been previously reported that Id1 and Id2 function as negative regulators of osteoclast differentiation^13^. To examine whether *Id1* and *Id2* are involved in the STAT5-mediated inhibition of osteoclast differentiation, STAT5A1*6 was overexpressed during osteoclast differentiation of the BMMs. Although this led to a significant increase in the mRNA expression of *Id1* and *Id2* (Fig. 4A), STAT5A1*6 did not affect the decrease in Id gene expression observed following RANKL stimulation. In both BMMs and pOC, the expression of *Id1* and *Id2*, but not that of *Id3*, was significantly increased in the presence of IL-3 (Fig. 4B). In STAT5-deficient cells, however, IL-3 failed to increase the expression of *Id1* and *Id2* (Fig. 4B), suggesting that IL-3 induces the expression of *Id1* and *Id2* through STAT5.

Next, we examined whether the ectopic expression of ID1 and ID2 could restore the IL-3-mediated inhibition of osteoclast differentiation that was abrogated by STAT5 deficiency. Consistent with our previous report^13^, ID1 or ID2 overexpression suppressed osteoclast development (data not shown), and the osteoclasts in STAT5-deficient cells were almost fully developed, even in the presence of IL-3 (Fig. 4C). Strikingly, quantification of the number of osteoclasts revealed that the osteoclast formation rescued from IL-3-mediated inhibition in STAT5-deficient cells was abolished by the ectopic expression of ID1 or ID2, where osteoclast differentiation was returned to almost the levels observed in the controls (Fig. 4C,D).

It has been reported that IL-3 induces dendritic cell differentiation^22^, and it therefore seemed possible that the increase in *Id1* and *Id2* gene expression by IL-3-mediated inhibition of osteoclast differentiation that occurs via STAT5 activation, as shown in the present study, was due to a change in the fate of the cells. In order to examine the effect of STAT5 activation on dendritic cell differentiation, dendritic cell surface markers, including CD80, CD86, MHC class II, and CD11c were analyzed during the osteoclast differentiation of BMMs overexpressing either an empty vector (pMX-IRES-EGFP) or the constitutively active from of STAT5A (STAT5A1*6) using fluorescence-activated cell sorting (FACS). Increase in the CD80+, CD86+, MHC class II+, and CD11c+ cell populations was observed in the presence of activated STAT5, indicating a conversion of cell fate from osteoclast precursors to dendritic cells (Supplementary Fig. 4). These results suggest that STAT5 activation by IL-3 inhibits RANKL-induced osteoclast differentiation via the induction of *Id1* and *Id2* expression, while converting the cell fate of osteoclast precursors to dendritic cells.

### STAT5 conditional knockout mice exhibit osteoporotic bone phenotype

**Figure 5:**
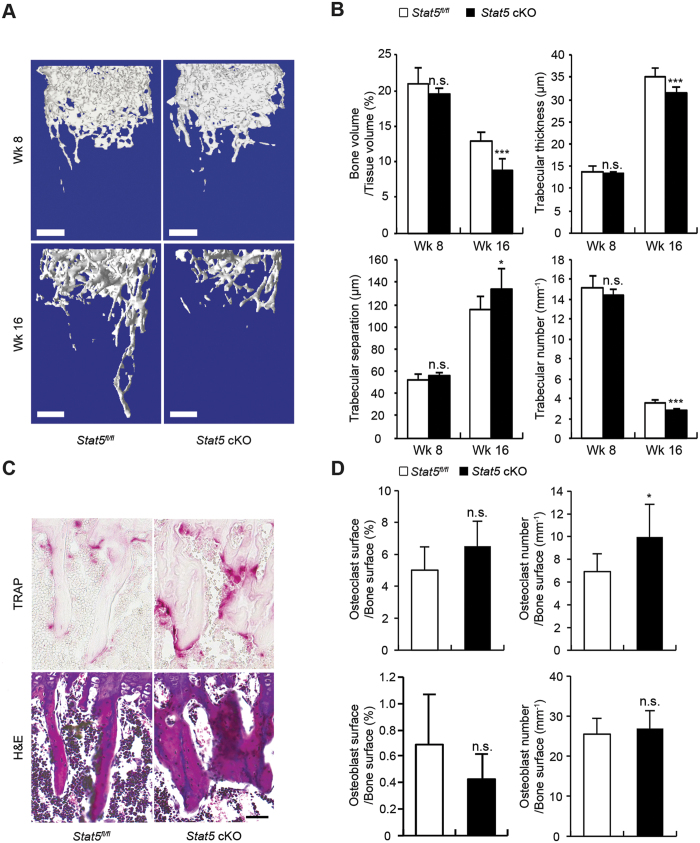
Bone phenotype of STAT5 conditional knockout mice. (**A**,**B**) Long bones obtained from 8-week-old and 16-week-old male *Stat5* cKO mice or *Stat5*^*fl/fl*^ littermates were subjected to μCT analysis. (**A**) Representative three-dimensional images of femurs in *Stat5* cKO mice or *Stat5*^*fl/fl*^ littermates. (**B**) Bone volume per tissue volume, trabecular bone thickness, trabecular separation and trabecular number were assessed from the μCT measurements. Data are represented as the mean ± SD. *P < 0.05; ***P < 0.001 vs. control; n.s., not significant; 8-week-old *Stat5*^*fl/fl*^, n = 5; 8-week-old *Stat5* cKO, n = 5; 16-week-old *Stat5*^*fl/fl*^, n = 9; 16-week-old *Stat5* cKO, n = 7. (**C**,**D**) Long bones obtained from 16-week-old male *Stat5* cKO mice or *Stat5*^*fl/fl*^ littermates were subjected to histomorphometric analyses. (**C**) Hematoxylin and eosin (H&E) and TRAP staining of histological sections of proximal tibiae. (**D**) Osteoclast surface per bone surface, osteoclast number per bone surface, osteoblast surface per bone surface, and osteoblast number per bone surface were assessed. Data are represented as the mean ± SD. *P < 0.05 vs. control; n.s., not significant; *Stat5*^*fl/fl*^, n = 7; *Stat5* cKO, n = 5. Bars: (**A**) 500 μm; (**C**) 100 μm. Statistical analyses were implemented in TTEST.

To evaluate the physiological functions of STAT5 in mice, the bone quality of the *Stat5*^*fl/fl*^ and *Stat5* cKO mice was compared. Microcomputed tomography (μCT) analysis with three-dimensional reconstruction of the trabecular bones of the distal femurs of 16-week-old male mice revealed a relatively lower bone mass in the *Stat5* cKO than in the *Stat5*^*fl/fl*^ mice, but there was no difference between 8-week-old *Stat5* cKO and *Stat5*^*fl/fl*^ mice (Fig. 5A). The reduced bone mass in 16-week-old *Stat5* cKO mice was accompanied by 32.8%, 10.6%, and 21.5% reductions in the bone volume, trabecular thickness, and trabecular numbers, respectively, and an increase of 15.8% in trabecular separation (Fig. 5B). Meanwhile, there was no significant difference in μCT parameters between *Stat5* cKO and *Stat5*^*fl/fl*^ mice at the age of 8 weeks (Fig. 5B). These results indicate that the *Stat5* cKO mice were more osteoporotic than the *Stat5*^*fl/fl*^ mice, and this effect was age-dependent. Furthermore, the number of osteoclasts and osteoblasts in the trabecular bones of the proximal tibia of the same mice at the age of 16 weeks analyzed with μCT were subjected to quantification using TRAP and H&E staining, respectively. The *Stat5* cKO mice exhibited a 5% increase in the number of osteoclasts and exhibited an increasing tendency without significance in the osteoclast surface. However, the number of osteoblasts in the trabecular bones was comparable between the *Stat5*^*fl/fl*^ and *Stat5* cKO mice, while the osteoblast surface exhibited decreasing tendency without significance (Fig. 5C,D), demonstrating that STAT5 deficiency in osteoclasts does not affect osteoblast differentiation. These results suggest that the reduced bone mass in the *Stat5* cKO mice is likely due to a reduced inhibitory effect of IL-3 on osteoclast differentiation via the STAT5-Id axis, which is different from that observed *in vitro*.

### Administration of IL-3 has no inhibitory effect on RANKL-induced bone destruction in STAT5 conditional knockout mice

**Figure 6:**
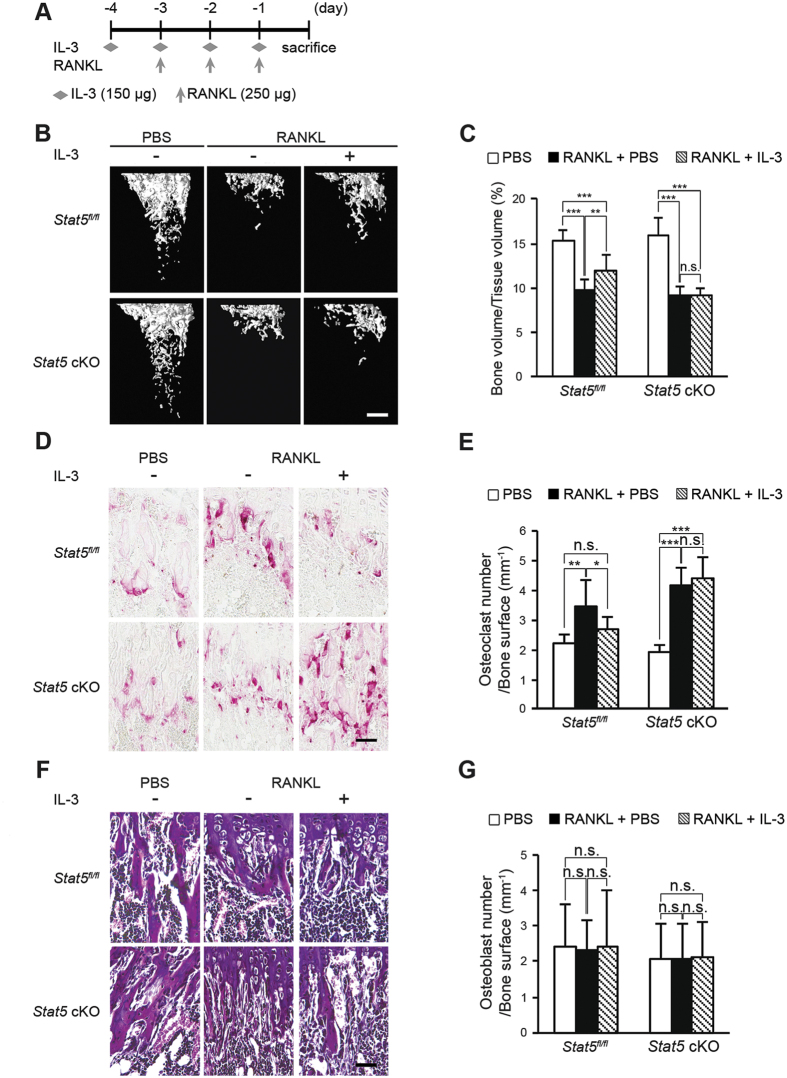
Administration of IL-3 reduces bone loss through STAT5 activation. (**A**–**G**) 8-week-old *Stat5* cKO mice or *Stat5*^*fl/fl*^ littermates were intraperitoneally administrated PBS, RANKL, with or without IL-3. Long bones obtained were subjected to μCT and histomorphometric analysis (**A**) Illustration of IL-3 administration strategy. (**B**) Representative three-dimensional images of femurs in *Stat5* cKO mice or *Stat5*^*fl/fl*^ littermates. (**C**) Bone volume per tissue volume was assessed from the μCT measurements. Data are represented as the mean ± SD. **P < 0.01; ***P < 0.001 vs. control; n.s., not significant; (PBS *Stat5*^*fl/fl*^ n = 8, *Stat5* cKO n = 8, RANKL *Stat5*^*fl/fl*^ n = 10, *Stat5* cKO n = 10, RANKL and IL-3 *Stat5*^*fl/fl*^ n = 14, *Stat5* cKO n = 11). (**D**) TRAP staining of histological section of proximal tibiae. (**E**) Osteoclast number per bone surface was assessed. Data are represented as the mean ± SD. ***P < 0.001 vs. control; **P < 0.01 vs. control; *P < 0.05 vs. control. n.s. not significant; n = 8. (**F**) Hematoxylin and eosin (H&E) stain of proximal tibiae. (**G**) Osteoblast number per bone surface was assessed. Data are represented as the mean ± SD. n.s., not significant; n = 8. Bars: (**B**) 500 μm; (**C**,**D**) 100 μm. All data were analyzed using ANOVA.

Having demonstrated the inhibitory effect of IL-3 on osteoclast differentiation, which occurs via STAT5 *in vitro*, we investigated the same effect *in vivo* using *Stat5*^*fl/fl*^ and *Stat5* cKO littermates following an intraperitoneal injection of RANKL with or without IL-3, as illustrated in Fig. 6A. μCT analysis of 8-week-old mice revealed that the bone volumes were reduced by 41.6% and 35.9% in the *Stat5* cKO and *Stat5*^*fl/fl*^ littermates, respectively, after RANKL injection. The bone loss observed in the RANKL-injected *Stat5*^*fl/fl*^ mice was recovered by 19.89% after intraperitoneal IL-3 administration. On the contrary, there was almost no bone volume recovery in the *Stat5* cKO mice, which continued to exhibit an osteoporotic phenotype, even after intraperitoneal IL-3 administration (Fig. 6B,C).

Next, histological analysis was performed to confirm bone loss recovery by intraperitoneal IL-3 administration at the cellular level in the tibiae of the *Stat5*^*fl/fl*^ and *Stat5* cKO littermate mice. After RANKL injection, there was an increase in the number of TRAP-positive cells, which indicated that the number of osteoclasts was increased by 116.09% and 54.78% in the *Stat5* cKO and *Stat5*^*fl/fl*^ littermates, respectively. Following IL-3 injection, the number of TRAP-positive cells was decreased by 22.41% in the *Stat5*^*fl/fl*^ mice, while there was no significant change in the osteoclast number in the *Stat5* cKO mice (Fig. 6D,E). In addition, there was no significant difference in the osteoblast numbers between the RANKL and IL-3-injected groups (Fig. 6F,G). These results indicate that bone loss by RANKL injection and bone mass recovery by IL-3 injection in the control mice were due to an increase and a decrease in the osteoclast numbers, respectively, rather than due to changes in the osteoblast numbers. However, the number of osteoclasts remained relatively high even when IL-3 was intraperitoneally administered in the *Stat5* cKO mice. In summary, IL-3 had an inhibitory effect on osteoclast differentiation *in vivo* in the presence of STAT5, whereas it failed to inhibit osteoclast differentiation in the absence of STAT5, indicating that STAT5 is a critical factor in the IL-3-mediated inhibition of osteoclast differentiation *in vivo* as well as *in vitro*.

## Discussion

This study showed that IL-3 negatively regulates RANKL-induced osteoclast differentiation through the transcription factors STAT5 and ID1/ID2 and shed light on the underlying mechanism. Although previous studies had reported that IL-3 inhibits human osteoclastogenesis and bone resorption through the downregulation of c-Fms^22^ and prevents RANKL-induced nuclear translocation of NF-κB^11^, the transcriptional circuit was not understood. Our study closes this gap and demonstrates that IL-3-induced STAT5 activation in osteoclast precursors reduces c-Fos expression and osteoclast differentiation. However, the induction of osteoclast differentiation by RANKL was independent of IL-3. Our study provides evidence indicating a direct contribution of STAT5 in the negative effect of IL-3 on osteoclast differentiation.

Previously, we had demonstrated that ID transcription factors are responsible for the negative regulation of RANKL-induced osteoclast differentiation^13^. In addition, IL-3-and STAT5-dependent activation of *Id1* transcription has been reported^23^. We chose to focus on IDs, as we believed that they might be directly responsible for the inhibition of osteoclast differentiation. Notably, the expression of *Id1* and *Id2*, but not that of *Id3*, was induced by IL-3 through STAT5 in the osteoclasts. In support of a critical function of ID1 or ID2, their overexpression significantly inhibited RANKL-induced osteoclast differentiation despite STAT5 deficiency. This indicates that IL-3 negatively regulates osteoclast differentiation through the activation of STAT5 and the subsequent induction of *Id1* and *Id2* expression. These findings are consistent with those of a previous study showing that IL-3 plays an inhibitory role in osteoclast differentiation by regulating the expression of c-Fos and Ids^14^.

Two independent studies suggested that IL-3 inhibits osteoclast differentiation by diverting the osteoclast precursor cells to either a macrophage or a dendritic cell lineage^11^,^22^. In the current study, we expanded on these findings and demonstrated that STAT5A1*6 overexpression increased the expression of dendritic cell markers, such as CD80, CD86, MHC class II, and CD11c. These findings solidify the notion that IL-3-induced STAT5 activation is responsible for the conversion of osteoclast precursor cells into dendritic cells.

Previously, Hirose *et al*. reported that conditional osteoclast-specific STAT5 mutant mice exhibited an osteoporotic phenotype^20^. Consistent with this, an osteoporotic bone phenotype was observed in the *Stat5* cKO mice in this study, indicating that STAT5 is essential for bone homeostasis under physiological conditions. While Hirose *et al*. demonstrated a significant increase in osteoclast surface but not osteoclast number the present study provided evidence of an increased osteoclast number but no significant change in osteoclast surface. These discrepancies are possibly because of the differences in the method used to delete STAT5 from the cells between the two studies. In this study, MX1-Cre was used to remove *Stat5* from hematopoietic stem cells and therefore STAT5 was absent from macrophage precursor cells from a very early stage, further confirming that STAT5 deficiency could mitigate cellular development, proliferation, and differentiation *in vivo*. In contrast, Hirose *et al*. utilized Cathepsin K-Cre to eliminate STAT5 in pre-osteoclast cells, which might have been more effective at a later stage, such as during resorption activity, than during cell proliferation and development. We suggest that the cell differential elimination of STAT5 elimination is a primary reason for the different lesions determined in these two studies.

It has been suggested that STAT5 deficiency increases the bone-resorbing activity of osteoclasts, which is related to the activation of ERK through the regulation of the expression of Dusp1 and Dusp2^20^. It is well known that ERK positively regulates osteoclast differentiation as well as bone resorptive activity^24^. Similarly, RANKL-induced ERK was slightly reduced upon overexpression of constitutively active STAT5 in the BMMs. Although further studies are needed to elucidate whether STAT5 activation regulates osteoclast differentiation and function by inhibiting RANKL-induced ERK activation, we propose that the reduced osteoclast differentiation caused by STAT5 activation is at least in part caused by decreased ERK activation.

The bone loss observed in the *Stat5* cKO mice seemed to be age-related. When the bone volumes of 8-week-old *Stat5* cKO and wild-type littermates were analyzed, there was no significant difference in bone volumes between the *Stat5* cKO and wild-type littermates. However, when the bone phenotype was analyzed between the *Stat5* cKO and wild-type littermates at the age of 16 weeks, the bone mass in the *Stat5* cKO mice was found to be reduced. Therefore, these observations imply that the bone loss observed in *Stat5* deficiency may be due to aging. In addition, the reduced bone mass observed in the absence of STAT5 at the age of 16 weeks appears to be associated with increased osteoclast differentiation rather than with the increased bone resorbing activity of osteoclasts. Although osteoclast differentiation remained unchanged when BMMs lacking STAT5 were cultured with RANKL, our *in vivo* study revealed a significant increase in the number of osteoclasts and a moderate increase in osteoclast surface. Collectively, these results suggest that STAT5 acts as a negative factor in osteoclast differentiation under the control of an existing endogenous signaling pathway *in vivo*, which may stimulate STAT5 activation and block osteoclast differentiation, rather than directly inhibiting the RANKL signaling pathway. IL-3 is a potent cytokine for the activation of STAT5 and the inhibition of osteoclast differentiation. Our results provided evidence suggesting that RANKL-induced bone loss was rescued by IL-3 administration only in the presence of STAT5. We hypothesize that RANKL increases IL-3 expression in osteoclasts and thus stimulates STAT5 activation, which ensures normal osteoclast differentiation and thus maintains bone homeostasis. In addition to IL-3, GM-CSF may contribute to bone homeostasis. Based on the *in vivo* mouse model, we propose that the IL-3/STAT5 pathway has a therapeutic value for age-related bone diseases.

## Methods

### Reagents

Recombinant human RANKL was purified from bacteria. Recombinant human M-CSF was a gift from Dr. Daved Fremont (Washington University, St. Louis, MO, USA). Recombinant mouse IL-3 and GM-CSF were purchased from R&D systems (Abingdon, UK).

### Mice

All animal experiments were approved by the Chonnam National University Medical School Research Institutional Animal Care and Use Committee and were carried out in accordance with the approved guidelines. STAT5 floxed (*Stat5*^*flox/flox*^) mice, targeting both *Stat5a* and *5b*, were generated as previously described^25^. To generate *Stat5* cKO mice (*Stat5*^*flox/flox*^;*Mx1-Cre*^+/‒^), STAT5^*fl/fl*^ mice were crossed with *Mx1-Cre*^+/‒^ mice expressing Cre recombinase under the control of a type I interferon-inducible Mx1 promoter. *Stat5*^*flox/flox*^ mice were crossed with *Mx1-Cre*^+/‒^ mice to produce *Stat5*^*flox*/+^;*Mx1-Cre*^+/‒^ offspring, which were subsequently backcrossed with *Stat5*^*flox/flox*^ mice to generate *Stat5* cKO mice. Genotyping was performed by PCR on tail genomic DNA with specific primers as follows; *Stat5 WT* forward, 5′-GAA AGC ATG AAA GGG TTG GAG-3′; *Stat5 WT* reverse, 5′-AGC AGC AAC CAG AGG ACT TAC-3′; *Stat5 fl2* forward, 5′-TAC CCG CTT CCA TTG CTC AG-3′; *Stat5 fl2* reverse, 5′-AGC AGC AAC CAG AGG ACT AC-3′; *Mx1-Cre* forward, 5′-GTG TTG CCG CGC CAT CTG C-3′; *Mx1-Cre* reverse, 5′-CAC CAT TGC CCC TGT TTC ACT ATC-3′. To induce *Stat5* excision by Cre expression, *STAT5*^*flox/flox*^;*Mx1-Cre*^+/‒^ mice were intraperitoneally injected with 0.25 mL of 1 mg/mL poly I:C (Sigma-Aldrich, St. Louis, MO, USA) three times (at two-day intervals) from postnatal day 10. *Stat5*^*flox/flox*^ littermates, that were identically administrated with poly I:C, were used as controls.

### Plasmid DNA constructs

The expression vector for constitutively active Stat5a (Stat5a1*6) has been described previously^26^. The full-length coding sequences of mouse Stat5a (NM_011488) and Stat5b (MN_011489) were purchased from 21C Frontier Human Gene Bank (Daejeon, Korea). The genes were subcloned into a retroviral vector (pMX-IRES-EGFP) that included a C-terminal Flag tag. Retrovirus vectors encoding ID1 and ID2 were described previously^13^.

### Cell cultures

Plat E cells were maintained in Dulbecco’s modified Eagle’s medium (DMEM) (HyClone Laboratories, Logan, UT, USA) supplemented with 10% FBS, puromycin (1 μg/mL) and blasticidin (10 μg/mL) every three days. Cells were plated onto either 100 mm dishes (1.3 × 10^6^ cell/dish) or 6-well plates (3 × 10^5^ cells/well) one day prior to transfection.

### Retroviral gene transduction

Plat E cells were transfected for retrovirus packaging with FuGENE 6 (Promega, Madison, WI, USA) according to the manufacturer instructions. Retroviral supernatant was collected from the culture media 48 hours after transfection. Murine bone marrow-derived macrophages (BMMs) were subsequently infected with the supernatant for eight hours in the presence of 10 μg/mL of polybrene (Sigma-Aldrich).

### Osteoclast formation

Osteoclasts were generated from murine bone marrow cells as previously described^27^. Bone marrow cells obtained via flushing the long bones of 6-week-old mice were cultured in α-MEM (HyClone Laboratories) containing 10% FBS, 100 U/mL penicillin and 100 mg/mL streptomycin (Life Technologies, Carlsbad, CA, USA) in the presence of M-CSF (30 ng/mL) for 3 days. After non-adherent cells were removed, adherent BMMs were plated onto 96-well plates (1 × 10^4^ cells/well) and further cultured with M-CSF (30 ng/mL) and RANKL (100 ng/mL). Cultured cells were fixed and stained for TRAP. TRAP-positive multinuclear cells (>3 nuclei/cell) [TRAP^+^ MNCs] were counted as osteoclasts. Cells were observed using a Leica DMIRB microscope equipped with an N Plan 10 × 0.25 numerical aperture objective lens (Leica Microsystems, Wetzler, Germany). Images were captured using ProgRes® Capture Pro software (Jenoptik, Jena, Germany).

### RT-PCR

Cells were lysed in Qiazol (QIAGEN GmbH, Hilden, Germany) and total RNA was isolated from cultured cells according to the manufacturer instructions. RNA (2 μg) was reverse transcribed into cDNA using Superscript II Reverse Transcriptase (Life Technologies). PCR with specific primers was used to determine mRNA expression level. Primer sequences were as follows: *Gapdh* forward, 5′-TGA CCA CAG TCC ATG CCA TCA CTG-3′; *Gapdh* reverse, 5′-CAG GAG ACA ACC TGG TCC TCA GTG-3′; *Stat5a* forward, 5′-AGT ATT ACA CTC CTG TAC TTG CGA AAG-3′; *Stat5a* reverse, 5′-GGA GCT TCT AGC GGA GGT GAA GAG ACC-3′; *Stat5b* forward, 5′-GGT CCC CTG TGA GCC CGC AAC-3′; *Stat5b* reverse, 5′-TGA CTG TGC GTG AGG GAT CCA CTG ACT-3′; *Nfatc1* forward, 5′-CTC GAA AGA CAG CAC TGG AGC AT-3′; *Nfatc1* reverse, 5′-CGG CTG CCT TCC GTC TCA TAG-3′.

### Real-time PCR

Quantitative real-time PCR analysis was performed in triplicate with SYBR Green (Qiagen) and with specific primers using Rotor-Gene Q (Qiagen). mRNA expression levels were normalized to *Gapdh*. The relative quantitation value for each target gene compared to the calibrator for that target was expressed as 2^−(Ct-Cc)^ (Ct and Cc are the mean threshold cycle differences after normalizing to *Gapdh*). The relative expression levels for each sample were presented by semi-log plot. Primer sequences were as follows: *Gapdh* forward, 5′-TGA CCA CAG TCC ATG CCA TCA CTG-3′; *Gapdh* reverse, 5′-CAG GAG ACA ACC TGG TCC TCA GTG-3′; *c-fos* forward, 5′-ATG GGC TCT CCT GTC AAC ACA CAG-3′; *c-fos* reverse, 5′-TGG CAA TCT CAG TCT GCA ACG CAG-3′; *Nfatc1* forward, 5′-CTC GAA AGA CAG CAC TGG AGC AT-3′; *Nfatc1* reverse, 5′-CGG CTG CCT TCC GTC TCA TAG-3′; *Acp5* forward, 5′-CTG GAG TGC ACG ATG CCA GCG ACA-3′; *Acp5* reverse, 5′-TCC GTG CTC GGC GAT GGA CCA GA-3′; *Oscar* forward, 5′-TGC TGG TAA CGG ATC AGC TCC CCA GA-3′; *Oscar* reverse, 5′-CCA AGG AGC CAG AAC CTT CGA AAC T-3′; *Id1* forward, 5′-AAG TGA GCA AGG TGG AGA TC-3′; *Id1* reverse, 5′-GGG CTG GAG TCC ATC TGG TC-3′; *Id2* forward, 5′-AAC ATG AAC GAC TGC TAC TC-3′; *Id2* reverse, 5′-AGA GTA CTT TGC TAT CAT TC-3′.

### Western blotting

Cells were washed with PBS and lysed in extraction buffer [50 mM Tris-HCl (pH 8.0), 150 mM NaCl, 1 mM EDTA, 0.5% Nonidet P-40, 1 mM PMSF and protease inhibitor cocktails]. Cell lysates were separated by SDS-PAGE and transferred to a PVDF membrane (Millipore corporation, Billerica, MA, USA). Membranes were blocked with 5% skim milk in TBS-T [10 mM Tri-HCl (pH 7.6), 150 mM NaCl, 0.1% Tween 20] and immunoblotted with antibodies against c-Fos (Santa Cruz Biotechnology, Dallas, TX, USA), NFATc1 (Santa Cruz Biotechnology), Phospho-STAT5 (Cell Signaling Technology), STAT5A (Cell Signaling Technology, Beverly, MA), IκB (Cell Signaling Technology), Flag-HRP (Sigma-Aldrich), Actin-HRP (Sigma-Aldrich), mouse-IgG-HRP (Abcam, Cambridge, UK) and rabbit-IgG-HRP (Abcam). Signals were detected with ECL solution (Millipore corporation) and analyzed using a LAS3000 luminescent image analyzer (GE Healthcare, Piscataway, NJ, USA).

### RNA sequencing

BMMs were isolated from *Stat5*^*fl/fl*^ and *Stat5* cKO littermates and differentiated to osteoclasts under the following conditions: *Stat5*^*fl/fl*^ BMMs with M-CSF (30 ng/mL) only; *Stat5*^*fl/fl*^ BMMs with M-CSF (30 ng/mL) and RANKL (100 ng/mL); *Stat5*^*fl/fl*^ BMMs with M-CSF (30 ng/mL), RANKL (100 ng/mL) and IL-3 (1 ng/mL); and *Stat5* cKO BMMs with M-CSF (30 ng/mL), RANKL (100 ng/mL) and IL-3 (1 ng/mL). Total RNA was extracted and mouse RNA Transcriptome Sequencing Analysis was performed using a HiSeq2000 system (Macrogen Inc., Korea). RNA sequencing data in this article have been deposited in Gene Expression Omnibus (GEO accession no. GSE76988).

### Micro-computed tomography (μCT)

Micro-computed tomography (μCT) images were taken using a high-resolution Skyscan 1172 system (Skyscan, Kontich, Belgium) with an X-ray source at 50 kV and 201 μA with a 0.5 mm aluminum filter. Images were captured every 0.7 degree angle with 11 μm·pixel^−1^. Raw images were reconstructed into serial cross-sectional images with identical thresholds (0 to 6,000 in Hounsfield units) for all samples using Recon software (Skyscan). A region of interest (ROI) was manually designated with CTAn software (Skyscan), within 300 steps of the trabecular bone of the distal femur, starting from 80 steps away from the epiphyseal plate. Three-dimensional morphometry was characterized by measuring the bone volume over the tissue volume (BV/TV), trabecular thickness (Tb.Th), trabecular separation (Tb.Sp), and trabecular number (Tb.N). Three-dimensional images of trabecular bones were remodeled with a window density from 90 to 165 using Ant software (Skyscan).

### Histomorphometric analysis

Tibiae collected from 8-week-old or 16-week-old mice were fixed overnight in 4% paraformaldehyde and decalcified in 5.5% EDTA in 3.8% formaldehyde buffer for two weeks at 4 °C. After samples were gradually dehydrated and embedded in paraffin, paraffin blocks were cut into 4 μm-thick longitudinal sections. Following deparaffinization with xylene, two consecutive sections were stained with tartrate-resistant acid phosphatase (TRAP) and hematoxylin & eosin (H&E) for determining osteoclasts and osteoblasts, respectively.

### Fluorescence-activated cell sorting

Bone marrow-derived macrophages were transduced with a control vector (pMX-FLAG-IRES-EGFP) or the constitutively active form of STAT5A (pMX-FLAG-IRES-EGFP-STAT5A1*6), differentiated to osteoclasts in the presence of M-CSF (30 ng/mL) and RANKL (100 ng/mL), and collected. The collected cells were stained with PE-anti-mouse CD80 (BD Pharmingen^TM^, San Diego, CA, USA), PE-anti-mouse CD 86 (BD Pharmingen^TM^), PE-anti-mouse MHC class II (eBioscience, San Diego, CA, USA), and APC-anti-mouse CD11c (eBioscience) for 20 min at 4 °C. Stained cells were analyzed using a Navios flow cytometer with Kaluza software (Beckman Coulter, Brea, CA, USA).

### Statistics

All values are expressed as the mean ± SD. Statistically significant differences were determined using a two-tailed Student’s *t*-test for two independent samples. Differences with a *P* value less than 0.05 were considered statistically significant. For statistical analyses of multiple group comparisons, analysis of variance (ANOVA) with post-hoc Tukey HSD test was performed to calculate *P* values. *P* < 0.05 was considered as statistically significant.

## Additional Information

**How to cite this article**: Lee, J. *et al*. STAT5 is a key transcription factor for IL-3-mediated inhibition of RANKL-induced osteoclastogenesis. *Sci. Rep*. **6**, 30977; doi: 10.1038/srep30977 (2016).

## Supplementary Material

Supplementary Information
