# Supplementary material for: STAT5 is a key transcription factor for IL-3-mediated inhibition of RANKL-induced osteoclastogenesis
Source: Sci Rep. 2016 Aug 3;6:30977. doi: 10.1038/srep30977 (PMC4971530; doi:10.1038/srep30977)
Supplement: Supplementary Information [file srep30977-s1.pdf]

STAT5 is a key transcription factor for IL-3-mediated inhibition of RANKL-induced osteoclastogenesis

Jongwon Lee[1,5], Semun Seong[1,2,5], Jung Ha Kim[1], Kabsun Kim[1], Inyoung Kim[1], Byung-chul Jeong[1,2], Kwang-Il Nam[3], Kyung Keun Kim[1], Lothar Hennighausen[4], Nacksung Kim[1,2,*]

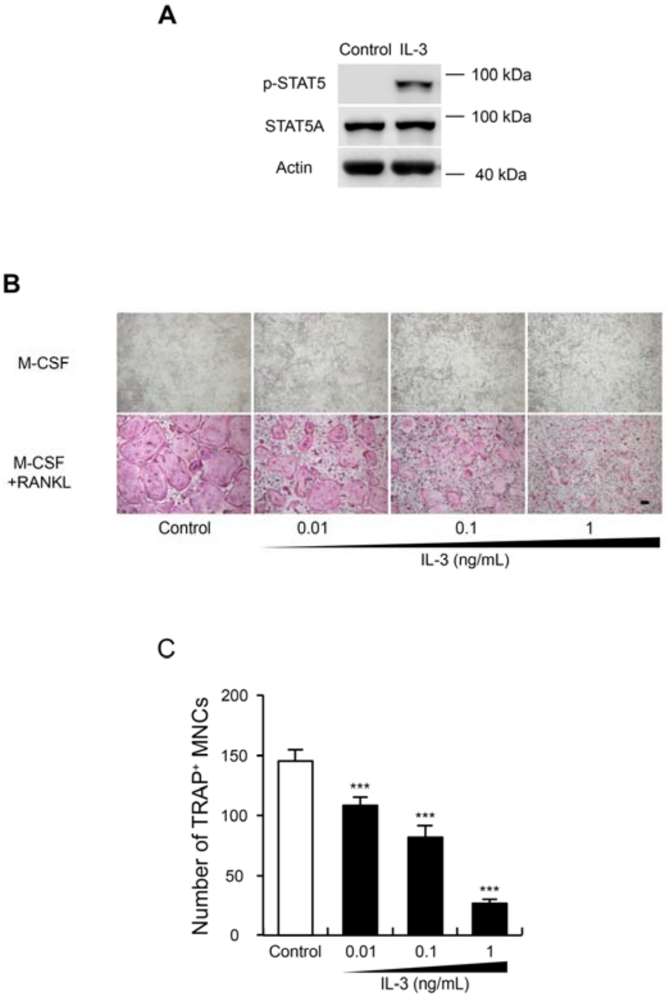
A
Control
IL-3
p-STAT5
STAT5A
Actin
100 kDa
100 kDa
40 kDa
B
M-CSF
M-CSF
+RANKL
Control
0.01
0.1
1
IL-3 (ng/mL)
C
Number of TRAP+ MNCs
200
150
100
50
0
***
***
***
Control
0.01
0.1
1
IL-3 (ng/mL)

Supplementary Figure 1. IL-3 inhibits RANKL-mediated osteoclastogenesis. (A) BMMs were cultured for one day with IL-3. Whole cell lysates were harvested from cultured cells and were immunoblotted with the indicated antibodies. (B-C) BMMs were cultured for three days with M-CSF and RANKL in the presence of various concentrations of IL-3 as indicated. (B) Cultured cells were stained for TRAP. (C) The number of TRAP-positive MNCs per well was counted. Data are represented as the mean ± SD. ***, $P < 0.001$ vs. control; $n = 4$. Bar: 100 μm. All results are representative of at least three independent experiments. Statistical analyses were performed using ANOVA.

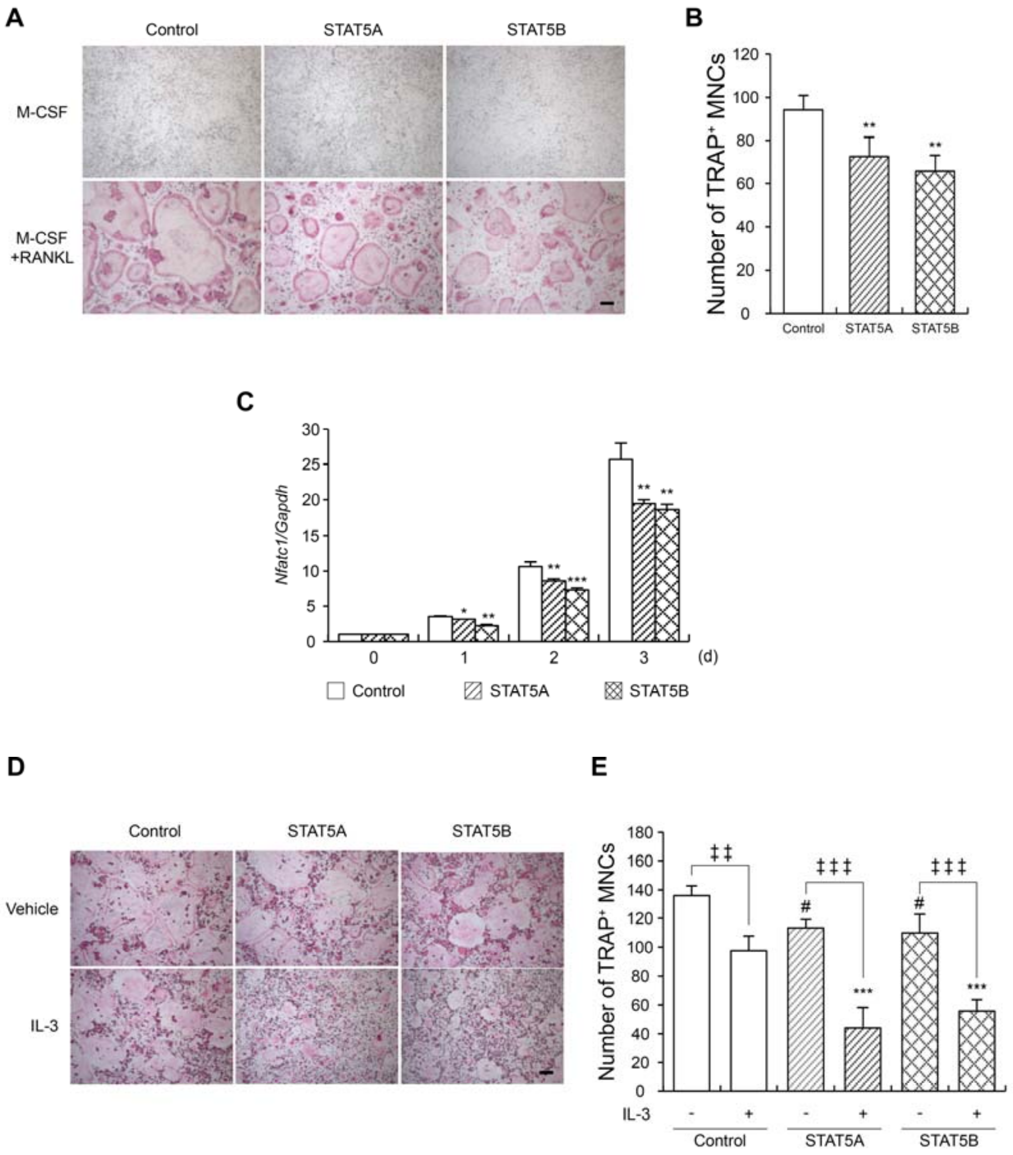
A
Control
STAT5A
STAT5B
M-CSF
M-CSF
+RANKL
B
Number of TRAP⁺ MNCs
Control
STAT5A
STAT5B
C
Nfatc1/Gapdh
(d)
Control
STAT5A
STAT5B
D
Control
STAT5A
STAT5B
Vehicle
IL-3
E
Number of TRAP⁺ MNCs
IL-3
Control
STAT5A
STAT5B

Supplementary Figure 2. Redundant roles of STAT5A and STAT5B in RANKL-induced osteoclastogenesis. (A-B) BMMs were transduced with pMX-IRES-EGFP (control), STAT5A, or STAT5B retrovirus and cultured with M-CSF for three days in the presence or absence of RANKL as indicated. (A) Cultured cells were stained for TRAP. (B) The number of TRAP-positive MNCs per well was counted. Data are represented as the mean ± SD. **, $P < 0.01$ vs. control; $n = 4$. (C) BMMs were transduced with pMX-IRES-EGFP (control), STAT5A, or STAT5B retrovirus and cultured with M-CSF and RANKL for the indicated times. mRNA levels of *Naftc1* were assessed by quantitative real-time PCR. Data represent mean ± SD of triplicate samples. *, $P < 0.05$; **, $P < 0.01$; ***, $P < 0.001$; vs. control. (D-E) BMMs were transduced with pMX-IRES-EGFP (control), STAT5A, or STAT5B retrovirus and cultured with M-CSF and RANKL for three days in the presence or absence of IL-3 as indicated. (D) Cultured cells were stained for TRAP. (E) The number of TRAP-positive MNCs per well was counted. Data are represented as the mean ± SD. #, $P < 0.05$; vs. IL-3-untreated control, ***, $P < 0.001$ vs. IL-3-treated control; ‡‡, $P < 0.01$; ‡‡‡, $P < 0.001$ versus the indicated lines; $n = 4$. Bars: (A and D) 100 μm. All results are representative of at least three independent experiments. All statistical analyses were performed using ANOVA.

A

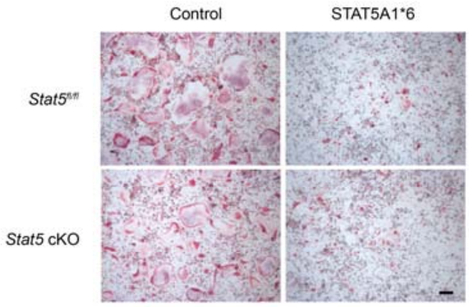

Control
STAT5A1*6
*Stat5*fl/fl
Stat5 cKO

B

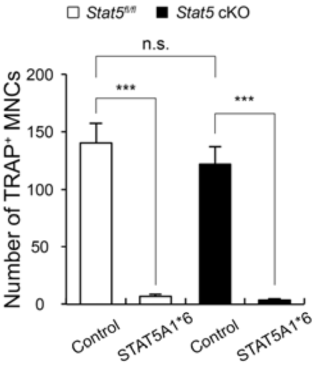

*Stat5*fl/fl
Stat5 cKO
n.s.
***
***
Number of TRAP+ MNCs
200
150
100
50
0
Control
STAT5A1*6
Control
STAT5A1*6

Supplementary Figure 3. STAT5A activation is sufficient to inhibit osteoclastogenesis independent of STAT5B. (A-B) Bone marrow cells were harvested from the long bones of *Stat5* cKO. BMMs were transduced with pMX-IRES-EGFP (control) or constitutively active STAT5A (STAT5A1*6) retrovirus and cultured with M-CSF and RANKL for three days. (A) Cultured cells were stained for TRAP. (B) The number of TRAP-positive MNCs per well was counted. Data are represented as the mean ± SD. ***, $P < 0.001$ vs. control;. n.s., not significant; $n = 4$. Bar: 100 μm. All results are representative of at least three independent experiments. Statistical analyses were performed using ANOVA.

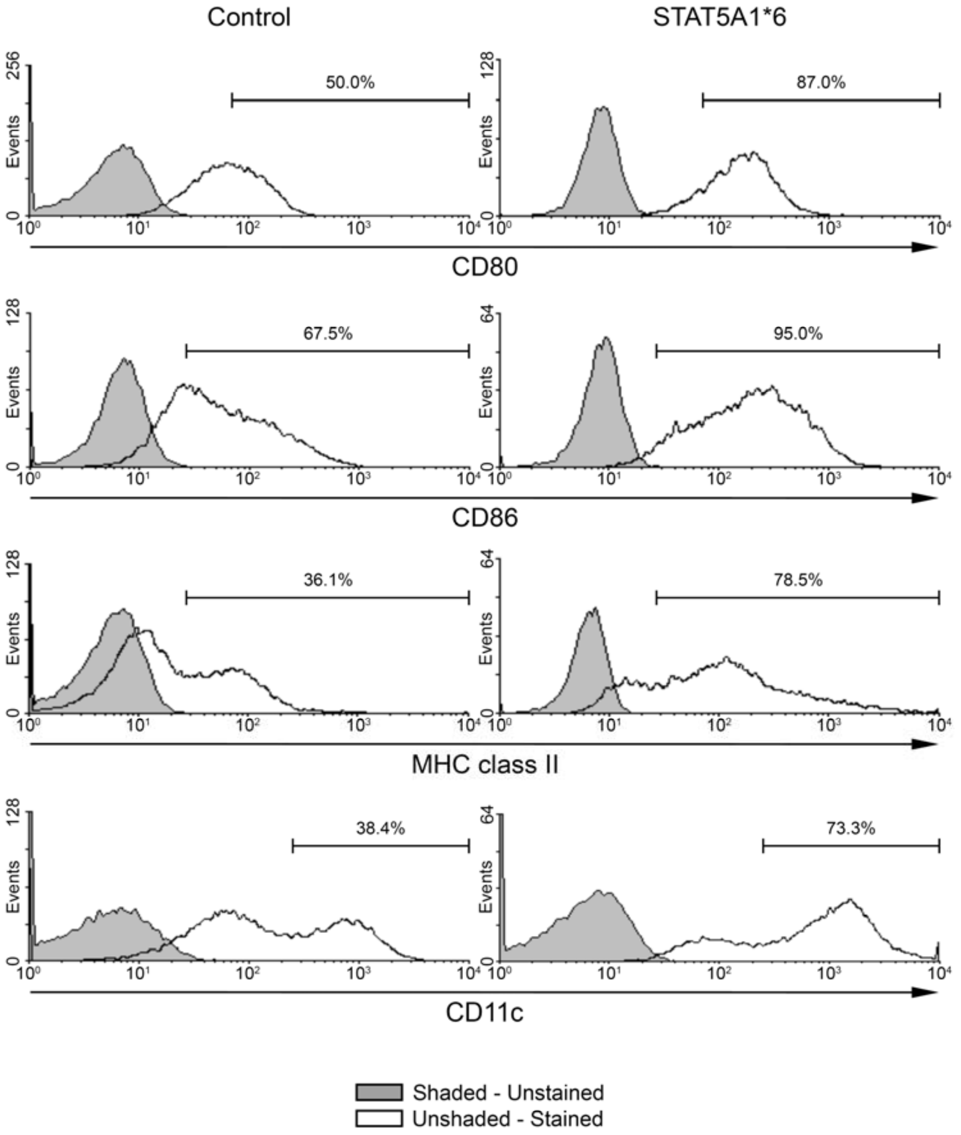

Control
STAT5A1*6
50.0%
87.0%
67.5%
95.0%
36.1%
78.5%
38.4%
73.3%
Events
CD80
CD86
MHC class II
CD11c
Shaded - Unstained
Unshaded - Stained

Supplementary Figure 4. Conversion of cell fate to dendritic cells by STAT5 activation. BMMs transduced with pMX-IRES-EGFP (control) or STAT5A1*6 retrovirus were cultured with M-CSF in the presence or absence of RANKL for three days and collected. The collected cells were stained with anti-CD80, anti-CD86, anti-MHC class II, and anti-CD11c antibodies and analyzed by fluorescence-activated cell sorting. Results are representative of at least three independent experiments.

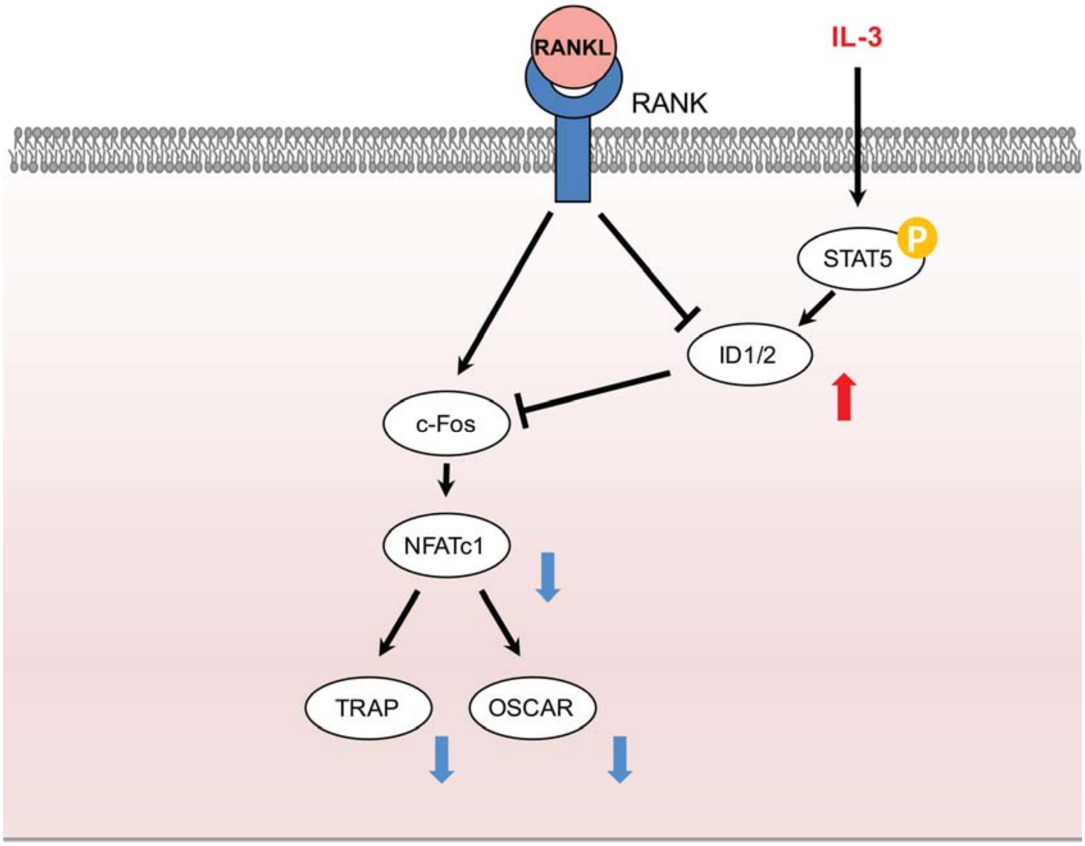
RANKL
RANK
IL-3
STAT5
P
ID1/2
c-Fos
NFATc1
TRAP
OSCAR

Supplementary Figure 5. Proposed model for the role of STAT5 in osteoclastogenesis. Summary of RANKL-induced osteoclast differentiation and the mechanism of IL-3-induced inhibition of osteoclast differentiation via STAT5-Id. Under normal physiological conditions, binding of RANKL to RANK induces c-Fos, which upregulates NFATc1, a master regulator of osteoclastogenesis, and its downstream signals such as *TRAP, OSCAR, etc*. This signaling pathway is interrupted when IL-3 phosphorylates STAT5 and the activated STAT5 induces ID1/2, which in turn downregulates c-Fos and its downstream signals, which ultimately results in attenuated osteoclastogenesis.

Supplementary Table 1. Gene expression changes in RANKL-mediated osteoclast differentiation and IL-3-mediated inhibition of osteoclast differentiation.

Category	Gene_symbol	Gene expression fold change		
		WT-M-CSF-RANKL vs. WT-M-CSF	WT-M-CSF-RANKL-IL-3 vs. WT-M-CSF-RANKL	KO-M-CSF-RANKL-IL-3 vs. WT-M-CSF-RANKL-IL-3
WT-M-CSF-RANKL vs. WT-M-CSF (down) and WT-M-CSF-RANKL-IL-3 vs. WT-M-CSF-RANKL (up) and KO-M-CSF-RANKL-IL-3 vs. WT-M-CSF-RANKL-IL-3 (down)	AA467197	**-3.204772**	**12.343473**	**-4.30564**
	Alox5	**-3.078875**	**4.268578**	**-3.558701**
	Ang	**-10.194463**	**3.650933**	**-3.570706**
	Anxa3	**-7.258497**	**7.393438**	**-3.915549**
	Arg1	**-7.277787**	**109.872002**	**-35.973683**
	Arhgap25	**-4.475555**	**5.153716**	**-3.801109**
	Atf3	**-9.616502**	**9.709122**	**-7.180086**
	Atp6v0a1	**-7.591318**	**11.568932**	**-5.660032**
	Atp6v0a1	**-5.363165**	**7.559801**	**-4.396774**
	AU022793	**-4.439623**	**21.839085**	**-10.29531**
	B430306N03Rik	**-7.39276**	**7.154513**	**-3.776136**
	Bcl2a1b	**-3.794083**	**19.245227**	**-21.721051**
	Bcl2l1	**-11.122434**	**8.210381**	**-4.482407**
	Bcl2l1	**-7.265129**	**8.66144**	**-4.158217**
	C1qa	**-15.040143**	**7.474966**	**-3.748695**
	C1qb	**-10.313852**	**4.929246**	**-6.239563**
	C1qc	**-10.231077**	**4.31271**	**-3.513921**
	Ccl12	**-22.223777**	**242.879248**	**-9.819019**
	Ccl2	**-18.075896**	**48.901042**	**-5.832743**
	Ccl3	**-3.749232**	**34.452754**	**-10.053547**
	Ccl4	**-19.927549**	**99.107158**	**-11.82831**
	Ccl6	**-11.64122**	**210.069574**	**-41.536871**
	Ccl7	**-31.820448**	**167.568698**	**-7.840487**
	Ccr2	**-5.553874**	**5.145877**	**-5.661242**
	Ccr5	**-5.765497**	**8.743835**	**-6.095364**
	Cd200r1	**-4.068562**	**8.353642**	**-5.847812**
	Cd300lf	**-11.746858**	**31.272067**	**-8.688648**
	Cd300lf	**-3.92102**	**9.421184**	**-6.913499**

	Cd84	**-3.410261**	**4.323763**	**-4.672291**
	Cd84	**-4.728823**	**5.625843**	**-4.144187**
	Cep250	**-4.258908**	**4.975471**	**-4.261925**
	Ch25h	**-21.620895**	**55.58655**	**-9.64555**
	Cmklr1	**-5.197711**	**9.885982**	**-6.128512**
	Cndp2	**-4.764804**	**6.412249**	**-3.08357**
	Cnr2	**-3.056071**	**4.968549**	**-4.435651**
	Ctsh	**-3.219848**	**3.460654**	**-4.48648**
	Dab2	**-9.529592**	**6.91415**	**-3.056564**
	Dnmt3l	**-4.611788**	**6.849288**	**-4.209885**
	Fam20c	**-4.029736**	**8.110167**	**-6.146261**
	Fcgr1	**-17.128146**	**11.836058**	**-4.039028**
	Fcgr3	**-8.976882**	**13.13273**	**-5.916132**
	Fn1	**-5.767593**	**5.898017**	**-7.024515**
	Fn1	**-3.477186**	**6.380904**	**-5.934579**
	Fn1	**-7.06525**	**5.197604**	**-4.593518**
	Fn1	**-4.860589**	**3.220314**	**-4.172795**
	Fn1	**-3.929027**	**5.326355**	**-4.112473**
	Gdf15	**-3.123662**	**5.359159**	**-3.419765**
	H2-DMa	**-3.484855**	**7.263071**	**-6.552643**
	Hal	**-9.458547**	**9.027426**	**-4.848071**
	Hlx	**-3.109511**	**4.429881**	**-3.01627**
	Hmga1	**-10.023472**	**11.778641**	**-12.018328**
	Hmox1	**-6.475946**	**5.084216**	**-3.143732**
	Icosl	**-6.749219**	**4.463312**	**-3.103888**
	Id1	**-15.145906**	**13.539643**	**-4.997802**
	Id2	**-5.797508**	**8.816544**	**-5.219676**
	Il21r	**-4.137183**	**6.138872**	**-4.157938**
	Irf8	**-9.328849**	**6.539895**	**-3.679706**
	Kctd12	**-4.057421**	**6.302394**	**-3.322494**
	Litaf	**-3.175425**	**4.610565**	**-3.620559**
	Maf	**-14.06373**	**6.633199**	**-3.309991**
	Mertk	**-5.174899**	**4.870496**	**-3.500171**
	Mgst1	**-5.063172**	**4.664832**	**-5.001095**
	Mrc1	**-5.35804**	**7.238886**	**-3.990227**
	Nxpe5	**-4.010395**	**5.358019**	**-4.344138**
	Pf4	**-6.063321**	**14.184867**	**-6.431475**
	Pilra	**-5.078432**	**8.439752**	**-3.362338**
	Plek	**-3.821644**	**5.455437**	**-3.00209**

	Psap	**-141.941266**	**215.840179**	**-5.744513**
	Ptgs1	**-3.681428**	**9.812489**	**-6.384626**
	Rab3il1	**-11.431955**	**7.209147**	**-3.72051**
	Rhob	**-3.837444**	**4.962606**	**-3.662089**
	Rnase6	**-4.37949**	**3.235004**	**-3.047195**
	Rnasel	**-3.32473**	**5.928657**	**-4.867796**
	Slc15a3	**-11.630782**	**15.919331**	**-5.335479**
	Slc36a1	**-3.053709**	**3.839491**	**-3.15042**
	Slc40a1	**-20.945115**	**16.528046**	**-7.577392**
	Tbxas1	**-4.306787**	**4.338054**	**-4.146963**
	Tlr1	**-3.200308**	**5.423097**	**-5.013597**
	Tlr8	**-7.31868**	**4.726743**	**-3.224056**
	Tmem132a	**-6.544304**	**9.374055**	**-4.865452**
	Tmem154	**-4.984457**	**9.923224**	**-7.001715**
	Tmem37	**-7.724976**	**3.205014**	**-3.092214**
	Trem1	**-6.133213**	**9.081651**	**-3.933309**
	Zranb3	**-9.780071**	**11.305611**	**-7.562593**
	Zyx	**-3.145585**	**5.469068**	**-3.70631**
**WT-M-CSF-RANKL vs. WT-M-CSF (up) and WT-M-CSF-RANKL-IL-3 vs. WT-M-CSF-RANKL (down) and KO-M-CSF-RANKL-IL-3 vs. WT-M-CSF-RANKL-IL-3 (up)**	Acp5	**72.542687**	**-23.308944**	**7.278308**
	Avpi1	**3.904183**	**-3.807859**	**3.261959**
	Bbs9	**8.022205**	**-3.888928**	**3.566136**
	Bdh2	**3.166562**	**-5.521662**	**3.284488**
	Cav1	**9.111685**	**-5.883492**	**3.200653**
	Cav1	**11.990228**	**-5.400171**	**3.150264**
	Cd82	**3.533061**	**-3.650205**	**4.674451**
	Ctsk	**75.454743**	**-12.80813**	**3.851024**
	Dusp9	**5.5049**	**-5.913806**	**3.457609**
	Fam83g	**5.089386**	**-4.276312**	**3.260021**
	Gfi1b	**5.56785**	**-4.007287**	**3.579352**
	Gfra2	**13.724001**	**-10.860133**	**6.551509**
	Gfra2	**5.637937**	**-3.772141**	**3.284024**
	Gja1	**3.778619**	**-6.978433**	**4.616275**
	Gm11545	**3.519081**	**-4.332652**	**4.313536**
	Gtf3c5	**6.60216**	**-3.923702**	**3.214448**
	Igsf11	**3.760873**	**-3.558916**	**3.011648**
	Jdp2	**32.548581**	**-12.319083**	**7.114027**
	Jdp2	**18.431914**	**-9.464058**	**6.51116**
	Lamc2	**5.095735**	**-4.490866**	**3.243346**
	Lphn2	**4.862458**	**-3.092864**	**3.395416**

	Mmp14	**12.89212**	**-5.249274**	**3.30948**
	Mmp9	**16.84516**	**-7.494702**	**4.071386**
	Mustn1	**6.606894**	**-4.886056**	**3.015609**
	Mycl	**16.905582**	**-6.923258**	**6.113919**
	Nfatc1	**97.372702**	**-37.164703**	**17.161056**
	Nfatc1	**8.535158**	**-5.425237**	**3.648573**
	Nolc1	**8.036622**	**-8.427702**	**9.31993**
	Nox1	**7.688506**	**-5.489285**	**3.776971**
	Nrp2	**8.551599**	**-7.50853**	**3.778248**
	Nrp2	**5.628992**	**-5.603937**	**3.638669**
	Oaf	**5.732848**	**-4.553084**	**4.792864**
	Ocstamp	**21.820044**	**-9.998031**	**5.50078**
	Osbpl9	**6.643192**	**-5.038208**	**3.099599**
	Pde2a	**5.531907**	**-3.553674**	**3.06715**
	Pltp	**3.018255**	**-5.121849**	**3.321738**
	Plxna4	**6.780524**	**-6.559659**	**4.438787**
	Prdm1	**12.782004**	**-9.138299**	**4.147898**
	Pus10	**3.098**	**-5.78979**	**3.700373**
	Rcan1	**8.290801**	**-8.117266**	**4.500374**
	Rgs16	**6.23316**	**-5.700509**	**51.281159**
	Scin	**25.013124**	**-13.967793**	**8.682338**
	Sdc1	**15.629892**	**-8.269206**	**5.258236**
	Sema7a	**5.372466**	**-4.519331**	**7.588241**
	Sept9	**13.08068**	**-5.534157**	**4.690844**
	Serpinb6b	**14.300244**	**-13.475148**	**9.80544**
	Slc6a4	**12.109968**	**-7.685788**	**5.530507**
	Slc9b2	**14.825507**	**-13.688978**	**5.257152**
	Tmem26	**3.573558**	**-3.452836**	**3.97436**
	Tsc22d1	**18.64694**	**-8.216998**	**4.434437**
	Vegfc	**15.72597**	**-6.670281**	**5.49483**
	Zbtb32	**7.81553**	**-5.929728**	**6.157381**
**and WT-M-CSF-RANKL-IL-3 vs. WT-M-CSF-RANKL (up) and**	Arrb2	**3.263433**	**2.311626**	**-7.586969**

	Cd274	**4.004952**	**2.099275**	**-3.169258**
	Il1rn	**6.946865**	**2.708472**	**-5.262722**
	Socs3	**5.847861**	**2.582227**	**-2.508697**
**WT-M-CSF-RANKL vs. WT-M-CSF (down) and WT-M-CSF-RANKL-IL-3 vs. WT-M-CSF-RANKL (down) and KO-M-CSF-RANKL-IL-3 vs. WT-M-CSF-RANKL-IL-3 (up)**	Cx3cr1	**-4.508868**	**-3.572496**	**4.553183**

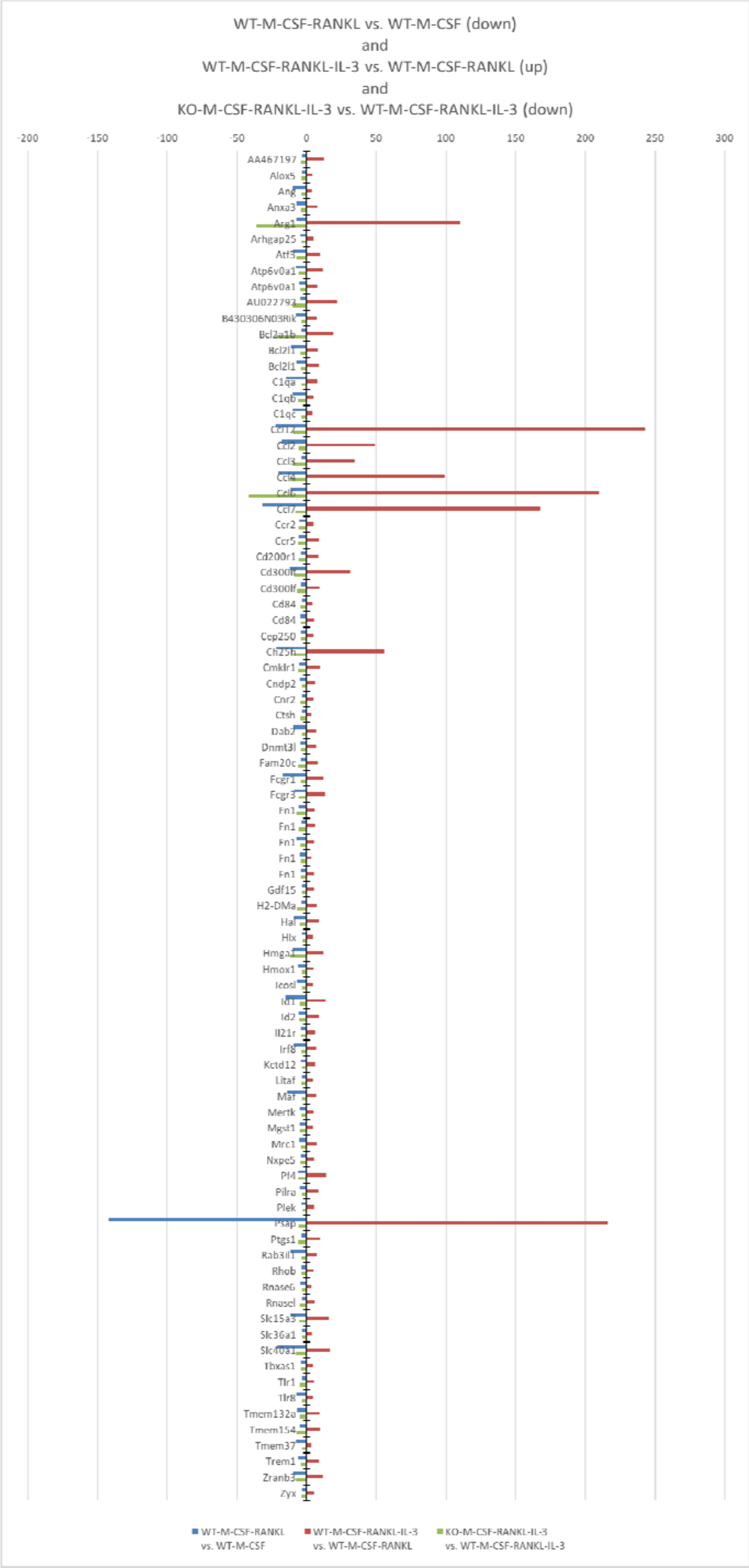

WT-M-CSF-RANKL vs. WT-M-CSF (down)
and
WT-M-CSF-RANKL-IL-3 vs. WT-M-CSF-RANKL (up)
and
KO-M-CSF-RANKL-IL-3 vs. WT-M-CSF-RANKL-IL-3 (down)
-200 -150 -100 -50 0 50 100 150 200 250 300
AA467197
Alox5
Ang
Anxa3
Arg1
Arhgap25
Atf3
Atp6v0a1
Atp6v0a1
AU022793
B430306N03Rik
Bcl2a1b
Bcl2l1
Bcl2l1
C1qa
C1qb
C1qc
Ccl12
Ccl2
Ccl3
Ccl4
Ccl6
Ccl7
Ccr2
Ccr5
Cd200r1
Cd300ld
Cd300lf
Cd84
Cd84
Cep250
Ch25h
Cmklr1
Cndp2
Cnr2
Ctsh
Dab2
Dnmt3l
Fam20c
Fcgr1
Fcgr3
Fn1
Fn1
Fn1
Fn1
Fn1
Gdf15
H2-DMa
Hal
Hlx
Hmga2
Hmox1
Icosl
Id1
Id2
Il21r
Irf8
Kctd12
Litaf
Maf
Mertk
Mgst1
Mrc1
Nxpe5
Pf4
Pilra
Plek
Psap
Ptgs1
Rab3il1
Rhob
Rnase6
Rnasel
Slc15a3
Slc36a1
Slc40a1
Tbxas1
Tlr1
Tlr8
Tmem132a
Tmem154
Tmem37
Trem1
Zranb3
Zyx
WT-M-CSF-RANKL vs. WT-M-CSF
WT-M-CSF-RANKL-IL-3 vs. WT-M-CSF-RANKL
KO-M-CSF-RANKL-IL-3 vs. WT-M-CSF-RANKL-IL-3

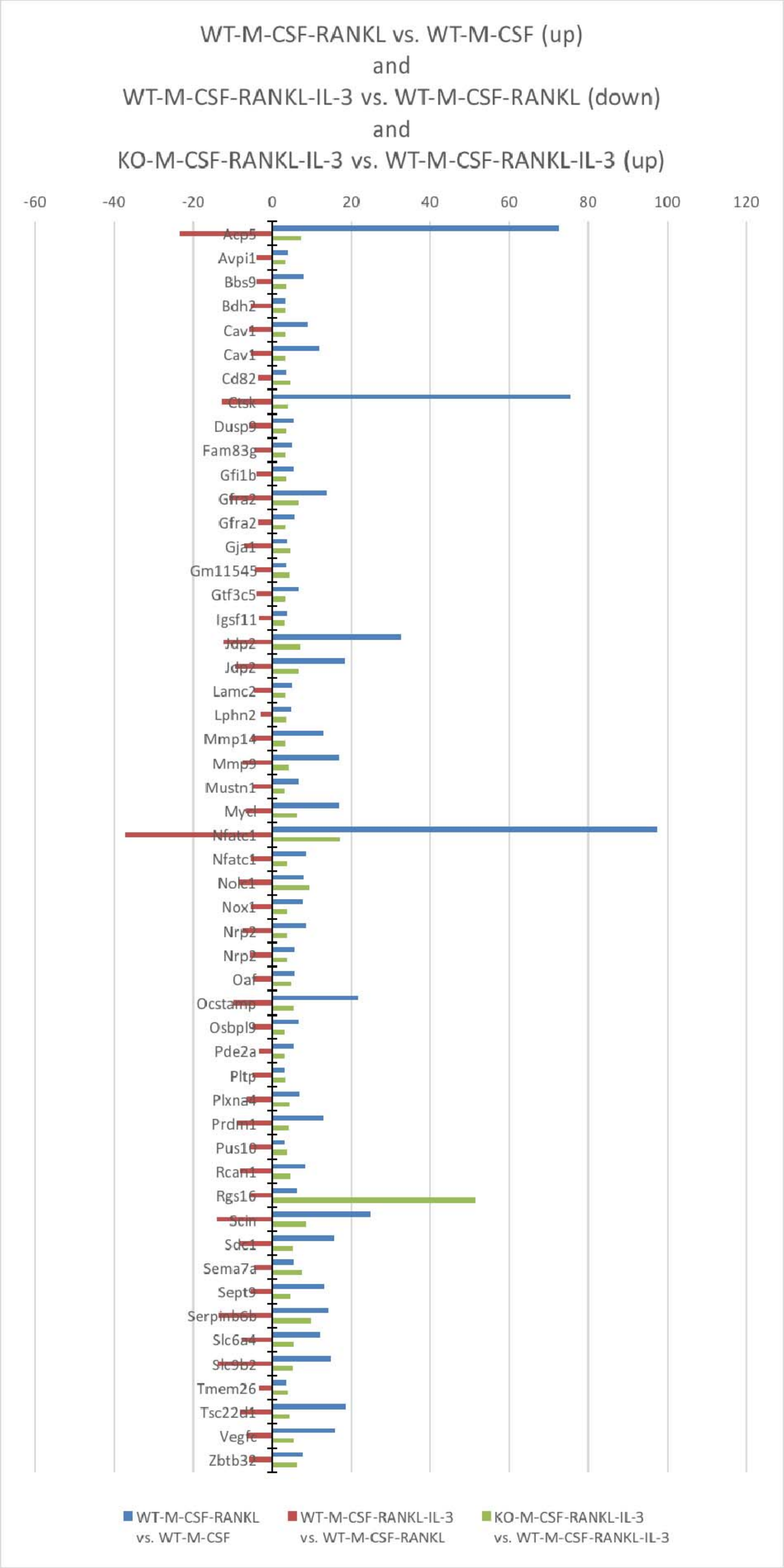

WT-M-CSF-RANKL vs. WT-M-CSF (up)
and
WT-M-CSF-RANKL-IL-3 vs. WT-M-CSF-RANKL (down)
and
KO-M-CSF-RANKL-IL-3 vs. WT-M-CSF-RANKL-IL-3 (up)
-60
-40
-20
0
20
40
60
80
100
120
Acp5
Avpi1
Bbs9
Bdh2
Cav1
Cav1
Cd82
Ctsk
Dusp9
Fam83g
Gfi1b
Gfra2
Gfra2
Gja1
Gm11545
Gtf3c5
Igsf11
Jdp2
Jdp2
Lamc2
Lphn2
Mmp14
Mmp9
Mustn1
Mycl
Nfatc1
Nfatc1
Nox1
Nox1
Nrp2
Nrp2
Oaf
Ocstamp
Osbpl9
Pde2a
Pltp
Plxna4
Prdm1
Pus10
Rcan1
Rgs16
Scin
Sdc1
Sema7a
Sept9
Serpinb6b
Slc6a4
Slc9b2
Tmem26
Tsc22d1
Vegfc
Zbtb32
WT-M-CSF-RANKL vs. WT-M-CSF
WT-M-CSF-RANKL-IL-3 vs. WT-M-CSF-RANKL
KO-M-CSF-RANKL-IL-3 vs. WT-M-CSF-RANKL-IL-3

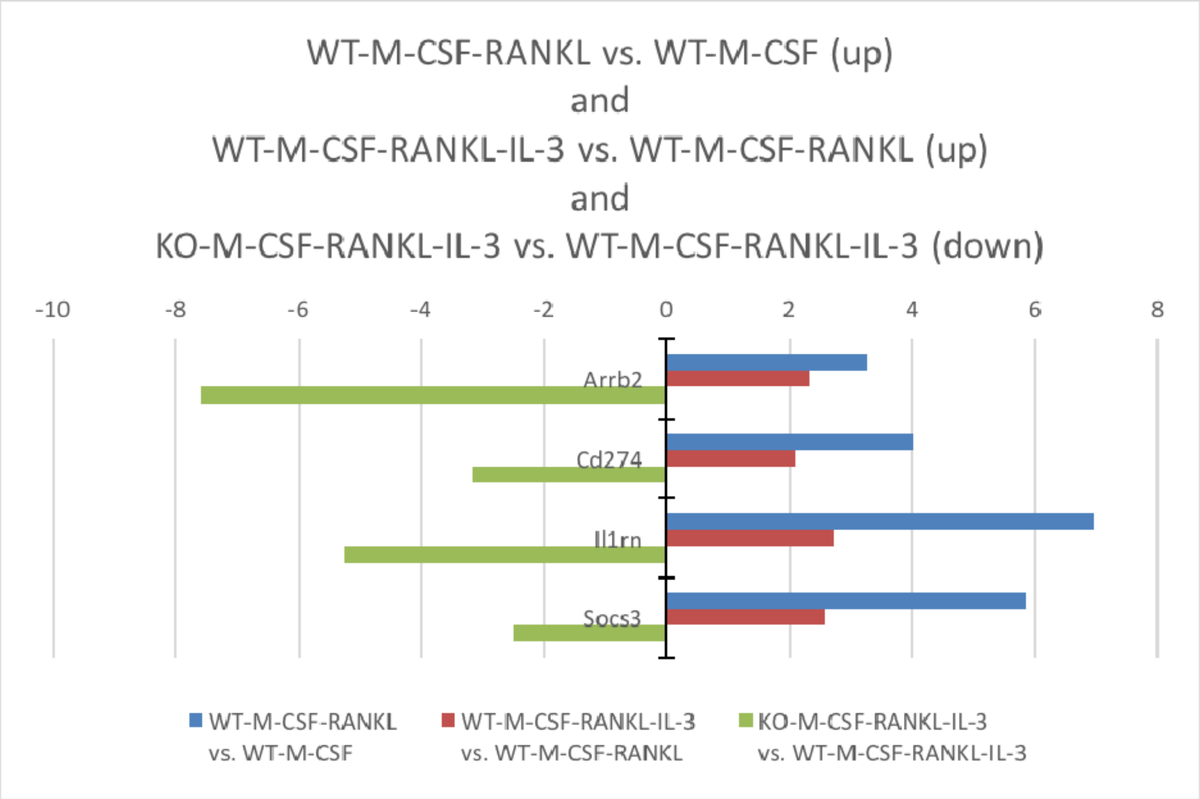
WT-M-CSF-RANKL vs. WT-M-CSF (up)
and
WT-M-CSF-RANKL-IL-3 vs. WT-M-CSF-RANKL (up)
and
KO-M-CSF-RANKL-IL-3 vs. WT-M-CSF-RANKL-IL-3 (down)
-10
-8
-6
-4
-2
0
2
4
6
8
Arrb2
Cd274
Il1rn
Socs3
WT-M-CSF-RANKL vs. WT-M-CSF
WT-M-CSF-RANKL-IL-3 vs. WT-M-CSF-RANKL
KO-M-CSF-RANKL-IL-3 vs. WT-M-CSF-RANKL-IL-3

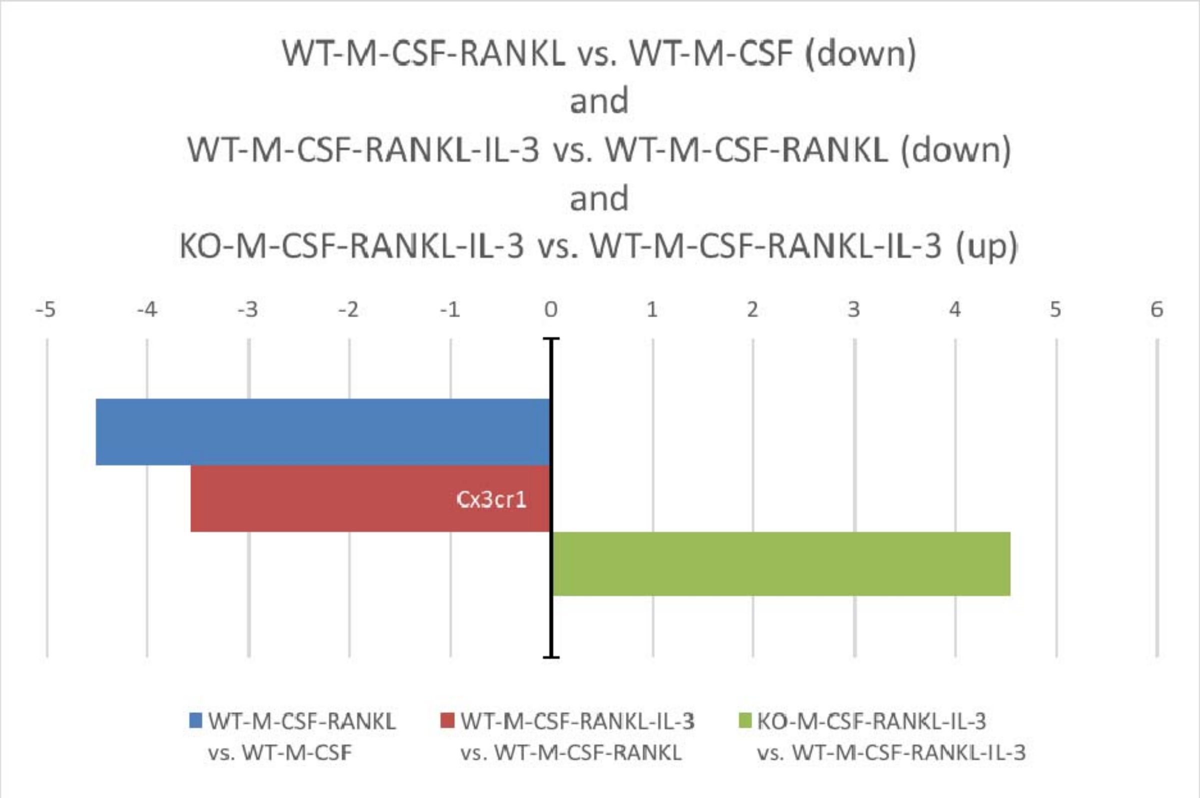
WT-M-CSF-RANKL vs. WT-M-CSF (down)
and
WT-M-CSF-RANKL-IL-3 vs. WT-M-CSF-RANKL (down)
and
KO-M-CSF-RANKL-IL-3 vs. WT-M-CSF-RANKL-IL-3 (up)
-5
-4
-3
-2
-1
0
1
2
3
4
5
6
Cx3cr1
WT-M-CSF-RANKL vs. WT-M-CSF
WT-M-CSF-RANKL-IL-3 vs. WT-M-CSF-RANKL
KO-M-CSF-RANKL-IL-3 vs. WT-M-CSF-RANKL-IL-3
